# Integrated metabarcoding and culturomics reveal the rot-suppressive rhizoplane mycobiome of cassava across contrasting agroecological habitats

**DOI:** 10.3389/fpls.2026.1782244

**Published:** 2026-08-05

**Authors:** Santha Divya, Tusar Kanti Bag, Muthulakshmi Lajapathy Jeeva, Thangaraj Makeshkumar, Binoy Ambika Manirajan, Vinod Chouhan, Syamala Swayamvaran Veena, Aundy Kumar

**Affiliations:** 1 Division of Plant Pathology, Indian Council of Agricultural Research (ICAR)–Indian Agricultural Research Institute, New Delhi, India; 2 Division of Crop Protection, Indian Council of Agricultural Research (ICAR)–Central Tuber Crops Research Institute, Thiruvananthapuram, Kerala, India; 3 School of Biosciences, Mahatma Gandhi University, Kottayam, Kerala, India

**Keywords:** biocontrol fungi, cassava stem and root rot, dysbiosis, *Fusarium falciforme*, rhizoplane mycobiome, wetland soils

## Abstract

**Introduction:**

Cassava stem and root rot caused by *Fusarium falciforme* (*Fusarium solani* species complex) constrains production in waterlogged tropical systems. In Kerala, India, disease endemicity differs markedly between wetlands and uplands, yet the ecological basis of this variation remains unresolved. We tested whether rhizoplane mycobiome structure and functional stability govern pathogen suppression across these habitats.

**Methods:**

Rhizoplane fungal communities from healthy and diseased plants in endemic wetlands and non-endemic uplands were profiled using Internal Transcribed Spacer amplicon sequencing, complemented by culture-dependent isolation and soil physicochemical analyses. Core taxa were identified through microbiome modeling and validated by ITS sequencing of isolates. Antagonistic activity against *F. falciforme* was assessed using dual-culture assays and greenhouse validation.

**Results:**

Quality filtering retained 847,760, 1,037,834, and 539,722 high-quality paired-end reads from the upland, wetland diseased, and wetland healthy rhizoplane samples, respectively. Healthy wetland rhizoplanes exhibited higher diversity and stability than diseased counterparts (Shannon +20%; inverse Simpson +60%). Disease was associated with diversity collapse and enrichment of opportunistic taxa, whereas upland communities remained distinct and stable. A conserved core mycobiome dominated by the members of *Pichia*, *Aspergillus*, *Sugiyamaella*, and *Fusarium* was identified, with *Penicillium* and *Talaromyces* forming an extended functional core. Isolates of *Penicillium oxalicum* and *Talaromyces pinophilus* inhibited *F. falciforme* by >90% *in vitro* and reduced disease incidence to 0% in greenhouse assays, matching chemical control. Soil acidity and nutrient imbalance were strongly associated with mycobiome disruption and disease.

**Discussion:**

Disease emergence coincided with loss of rhizoplane diversity and depletion of key antagonists, indicating failure of microbial resistance under edaphic stress. The consistent identification and validation of *P. oxalicum* and *T. pinophilus* as keystone antagonists support their functional role in pathogen suppression. Integrating microbiome conservation with soil health restoration offers a robust, climate-resilient strategy for managing cassava stem and root rot in tropical agroecosystems.

## Highlights

- Healthy wetland and upland rhizoplanes maintained balanced fungal communities likely contributing to disease suppression.
- Diseased wetland rhizoplanes showed reduced fungal diversity and disrupted community structure compared to healthy systems.
- A conserved core mycobiome was identified across habitats, with *Pichia* (100% prevalence) and an extended functional core including *Penicillium* and *Talaromyces*.
- Sequence-validated isolates of *Penicillium oxalicum* and *Talaromyces pinophilus* exhibited strong antagonism, suppressing *Fusarium falciforme* by up to 92.6% *in vitro*.
- Greenhouse assays confirmed complete disease suppression and significant growth promotion in cassava under pathogen pressure by both isolates.

## 1 Introduction

Cassava (*Manihot esculenta* Crantz) is a major tropical tuber crop and a key source of dietary energy for more than 500 million people, particularly in developing regions (Karlström et al., 2016; Otekunrin, 2024). Owing to its high starch content and caloric productivity, cassava ranks fifth among global food crops after maize, rice, wheat, and potato (FAO, 2022), and also supports a range of industrial applications, including starch and bioethanol production (Scaria et al., 2024). Its ability to grow in marginal soils with low inputs and tolerate climatic stress makes it a highly climate-resilient crop of considerable socio-economic importance to smallholder farmers (Immanuel et al., 2024; Borku et al., 2025; Bacsi and Jarso, 2026). In India, cassava is primarily cultivated in Kerala and Tamil Nadu, which together account for about 1.66 lakh hectares and 59.38 lakh tonnes of production, with Kerala contributing nearly 40% of the national output (GoI, 2023). Despite this, yields remain far below the potential of ~80 t ha^-1^, largely due to biotic constraints (McCallum et al., 2017; Alonso Chavez et al., 2022).

Cassava root rot disease (CRRD) is one of the most damaging biotic stresses, causing yield losses of up to 100% in susceptible cultivars (Hohenfeld et al., 2022). The disease affects tuberous roots and is caused by a complex of soilborne pathogens, predominantly species of *Fusarium*, *Phytophthora*, *Lasiodiplodia*, *Neoscytalidium*, and other associated fungi, with the pathogen composition varying across geographical regions (Boas et al., 2017; Silva et al., 2022; Sangpueak et al., 2023; da Silva et al., 2025; Subhash et al., 2025). In Kerala, cassava stem and root rot (CSRR) has intensified in wetland ecosystems, particularly following severe flooding events in 2020, leading to substantial crop losses (Jeeva et al., 2020). Members of the *Fusarium solani* species complex, especially *Fusarium falciforme*, have been identified as the primary causal agents (Jeeva et al., 2025).

Plant-associated microbial communities play a central role in nutrient cycling, stress tolerance, and disease suppression (Compant et al., 2019; Berg et al., 2020; Yin, 2026). Advances in high-throughput sequencing have greatly improved our understanding of microbial diversity across plant compartments, highlighting the importance of root-associated microbiota in host defence and pathogen suppression (Lareen et al., 2016; Liu et al., 2019; Teixeira et al., 2019; Du et al., 2025). Culture-independent approaches such as metagenomics have further revolutionized microbial ecology by revealing previously uncultured diversity and improving interpretation of plant-associated microbiomes, thereby deepening insights into ecosystem functioning (Parray et al., 2023; Chaudhari et al., 2023). While the rhizosphere has been widely studied, the rhizoplane-the microbial community directly associated with the root surface-remains relatively underexplored, despite its critical role as a selective interface at the plant–soil boundary that shapes root-associated microbial assembly (Bulgarelli et al., 2013; Edwards et al., 2015; Iqbal et al., 2024). The concept of a core microbiome, defined as taxa consistently associated with a host across environments, provides a useful framework for understanding microbial stability and functional resilience, with native core microbiomes increasingly recognized for their role in supporting plant-associated functions and plant performance (Shade and Handelsman, 2012; Zhou et al., 2024).

Conventional disease management strategies are often ineffective in waterlogged systems, where prolonged soil saturation favours pathogen survival and proliferation (McCallum et al., 2017). Microbiome-based approaches therefore offer a promising alternative, as beneficial microbes can enhance plant resilience and suppress pathogens (Trivedi et al., 2020). The contrasting disease dynamics observed between endemic wetlands and relatively suppressive upland systems provide an opportunity to identify fungal taxa associated with cassava health. In this study, we characterized the cassava rhizoplane mycobiome across these contrasting agroecological habitats using high-throughput amplicon sequencing. Specifically, we (i) compared fungal diversity and community structure between wetlands and uplands, (ii) identified core rhizoplane fungal taxa, (iii) validated sequencing results using culture-dependent methods, and (iv) assessed the antagonistic potential of core isolates against *F. falciforme*. Together, these findings provide a microbiome-mediated framework for developing sustainable and climate-resilient strategies to manage cassava stem and root rot.

## 2 Materials and methods

### 2.1 Study sites and rhizoplane sampling

The cassava rhizoplane mycobiome was profiled using high-throughput amplicon sequencing of the fungal internal transcribed spacer (ITS) region (Schoch et al., 2012). Sampling was conducted in December 2024 from a locally cultivated cassava variety, highly susceptible to stem and root rot. Two contrasting agroecological habitats in Thiruvananthapuram district, Kerala, India, were selected: a root rot–endemic wetland at Venganoor (8.408601 N, 76.989631 E; 44 m asl) and a non-endemic upland at Neyyattinkara (8.383752 N, 76.998306 E; 26 m asl). The wetland site was chosen based on its history of severe disease incidence and post-flooding losses, whereas the upland site represented a stable, non-endemic system cultivating comparable cassava varieties.

Root samples were collected from eight-month-old plants classified as healthy or diseased. Diseased plants were identified based on typical symptoms, including yellowing of leaves, collar rot, tuber and root rot, vascular discoloration, root decay, wilting, and reduced vigour. Disease status was confirmed by isolating pathogens on potato dextrose agar (PDA), followed by morphological and ITS-based molecular identification of *Fusarium falciforme* (GenBank accession PV982217.1).Three biological replicates per treatment were collected, each comprising pooled roots from 3–5 neighbouring plants. Roots were gently washed, transferred aseptically to sterile tubes, and transported at 4 ± 1 °C for further processing.

### 2.2 Soil physicochemical characterization

Soil samples from both sites were analysed using standard protocols. Soil pH and electrical conductivity were measured in a 1:2.5 soil–water suspension (Jackson, 1973). Organic carbon was determined using the Walkley and Black method (Walkley and Black, 1934), and available nitrogen by alkaline KMnO_4_ oxidation (Subbiah and Asija, 1956). Available phosphorus and potassium were measured using Bray No. 1 extraction (Bray and Kurtz, 1945) and ammonium acetate extraction followed by flame photometry, respectively (Jackson, 1973). Exchangeable calcium and magnesium were quantified by EDTA titration. Available sulphur was estimated by CaCl_2_ extraction (Massoumi and Cornfield, 1963), boron by hot water extraction with azomethine-H detection (Gupta, 1967), and micronutrients (Fe, Mn, Zn, Cu) by 0.1 N HCl extraction and atomic absorption spectrophotometry (Sims and Johnson, 1991).

### 2.3 DNA extraction, ITS metabarcoding, and taxonomic assignment

Rhizoplane fungi were detached by washing 10 g of roots in sterile phosphate-buffered saline containing 0.1% Tween-20 (PBST) with agitation at 250 rpm for 30 min at 28 °C, followed by vortexing. This step was repeated six times to obtain a composite suspension. Removal of surface microbes was verified through sterility checks. Microbial pellets were recovered by centrifugation at 12,000 × g for 30 min at 4 °C.

Genomic DNA was extracted using the NucleoSpin^®^ Soil Kit (Macherey-Nagel, Germany), quantified using a NanoDrop Lite Plus (Thermo Fisher Scientific), and checked on 1% agarose gels. Extraction blanks and no-template PCR controls were included to monitor contamination and were evaluated only by agarose gel electrophoresis. Because no visible amplification products were detected in these controls, they were not subjected to downstream library preparation or high-throughput sequencing.

The fungal ITS region was amplified using ITS1 and ITS4 primers with Illumina overhang adapters. PCR was performed using HotStar HiFidelity KAPA Master Mix in a Veriti™ Pro Thermal Cycler. Amplicons were purified, indexed with dual barcodes, quantified using a Qubit™ 4 Fluorometer, and validated on an Agilent TapeStation 4150. Libraries were pooled equimolarly and sequenced on an Illumina NextSeq 2000 platform (300 bp paired-end reads).

Raw sequencing reads were quality-assessed using FastQC v0.11.9 (Wingett and Andrews, 2018) and processed with fastp v0.23.2 for adapter trimming and quality filtering (Chen et al., 2018). Taxonomic classification was carried out using Kraken2 v2.1.2 (Wood et al., 2019), followed by species-level abundance estimation with Bracken v2.7.

### 2.4 Community composition and diversity analyses

Taxonomic summaries and relative abundance visualizations were generated in R version 4.2.2 using ggplot2 v3.4.0 (Wickham, 2016). Alpha and beta diversity metrics were calculated using phyloseq v1.42.0 (McMurdie and Holmes, 2013) and vegan v2.6-4 (Oksanen et al., 2025). Alpha diversity was evaluated using Chao1, ACE, Shannon, Simpson, Fisher's alpha, and observed richness indices. The analyses were further supported using MicrobiomeAnalyst 2.0 (Dhariwal et al., 2017) and RAISINS (R and AI Solutions for Inferential Statistics), an R-based cloud computing platform that integrates standard R statistical packages for ecological and inferential analyses (Hisham et al., 2025), available at https://raisins.live.

To account for differences in sequencing depth, data were normalized using total sum scaling (TSS). Rarefaction curves indicated adequate sequencing coverage across samples. Beta diversity was assessed using principal coordinates analysis (PCoA) based on Bray–Curtis dissimilarities. Statistical significance was evaluated using PERMANOVA (999 permutations), and homogeneity of group dispersion was tested using PERMDISP. P-values were adjusted using the Benjamini–Hochberg false discovery rate (FDR) correction.

### 2.5 Core mycobiome and shared OTU analysis

Core mycobiome analysis was performed on TSS-normalized data using MicrobiomeAnalyst 2.0. Core taxa were defined using a prevalence threshold of ≥70% across samples and a minimum relative abundance of 0.01%, ensuring the inclusion of consistently abundant and ecologically relevant taxa. Shared and unique operational taxonomic units (OTUs) were visualized using UpSetR (version 2022.12.0) (Lex et al., 2014), with data processing carried out using readxl, dplyr, and tidyr.

### 2.6 Culture-dependent isolation and taxonomic validation

Rhizoplane suspensions were also subjected to culture-dependent analysis. Serial dilutions were plated on potato dextrose agar and Rose Bengal agar and incubated at 28 ± 2 °C. Representative colonies were purified, morphologically characterized, and maintained on PDA slants at 4 °C. Genomic DNA was extracted using the CTAB method (Moore et al., 2004). The ITS region was amplified using ITS1 and ITS4 primers and sequenced (Raja et al., 2017). Sequences were identified using BLASTn against the NCBI GenBank database (≥97% similarity), deposited in GenBank, and cross-validated with amplicon-derived taxa.

### 2.7 Antagonistic activity against *Fusarium falciforme*

*Fusarium falciforme* (GenBank accession PV982217.1), isolated from diseased cassava plants collected during the study and maintained at ICAR–CTCRI, Thiruvananthapuram, was cultured on potato dextrose agar (PDA) for subsequent experiments. Antagonistic activity of rhizoplane fungal isolates was evaluated using a dual culture assay (Skidmore and Dickinson, 1976). Mycelial discs of the pathogen and antagonist were placed opposite each other on PDA plates and incubated at 25–28 °C for seven days. Radial growth inhibition was calculated using Vincent’s formula (Vincent, 1947). Each assay included three replicates and was conducted twice.

### 2.8 *In planta* evaluation of antagonistic core fungal isolates under greenhouse conditions

#### 2.8.1 Pathogen inoculum preparation and soil infestation

*Fusarium falciforme* was mass-multiplied on a sand–maize meal substrate following Kala et al. (2016). Briefly, 900 g of washed river sand was mixed with 100 g maize meal and 200 mL distilled water in 1 L conical flasks. The mixture was autoclaved, inoculated with actively growing mycelial plugs from PDA cultures, and incubated at 28 ± 2 °C for two weeks to obtain fully colonized inoculum.

#### 2.8.2 Preparation and application of fungal antagonists

The most effective antagonistic isolates identified *in vitro*-*Penicillium oxalicum* 1WHF_44 and *Talaromyces pinophilus* ULF_15-were evaluated under greenhouse conditions, along with additional isolates (*Trichoderma asperellum* WDF6_40 and *Trichoderma* sp. WHF_43). A recommended *Trichoderma asperellum* isolate from CTCRI was included as a biological reference control.

Fungal inocula were prepared from actively growing PDA cultures and mass-multiplied on sterilized sorghum grains at 28 ± 2 °C for 12 days. The colonized grains were further propagated in sterilized farmyard manure (FYM) and incorporated into the potting medium at 50 g kg^-1^ soil at planting to promote rhizosphere establishment, following established methods for *Trichoderma* spp (Rini and Sulochana, 2007). Cassava setts were dipped in spore suspensions (1 × 10^6^ spores mL^-1^) for 30 min prior to planting.

#### 2.8.3 Greenhouse experiment

The experiment was conducted in a greenhouse using a completely randomized design with eight treatments and ten replications per treatment. A locally cultivated cassava variety highly susceptible to stem and root rot was used. Stem cuttings (15–20 cm long, six nodes) were planted in plastic pots (17 cm diameter × 14.5 cm height) containing ~2 kg of sterilized soil and FYM (50:1, w/w).

For pathogen challenge treatments, *F. falciforme* inoculum was incorporated into the potting mixture at a 1:10 ratio (w/w) prior to planting (Subhash et al., 2025). Treatments included combinations of pathogen with each antagonist (*P. oxalicum* 1WHF_44, *T. pinophilus* ULF_15, *T. asperellum* WDF6_40, *Trichoderma* sp. WHF_43, and the CTCRI *T. asperellum* isolate), along with a chemical control (carbendazim 50% WP at 0.1%), a pathogen-inoculated control, and an uninoculated absolute control.

Disease incidence was assessed based on collar rot and wilting symptoms. At harvest, plant height, number of leaves, shoot weight, sett weight, root weight, root number, and root length were recorded. Data were analysed using analysis of variance (ANOVA) in RAISINS (R and AI Solutions for Inferential Statistics) (Hisham et al., 2025). Means were separated using the least significant difference (LSD) test at p ≤ 0.05, and effect size was estimated using Cohen’s *F*.

### 2.9 Data accessibility

All raw sequencing reads have been deposited in the NCBI Sequence Read Archive under accession number PRJNA1391667.

## 3 Results

### 3.1 Metabarcoding output, data quality, and rhizoplane mycobiome diversity

ITS amplicon sequencing generated a total of 861,322, 1,045,778, and 548,278 paired-end reads from upland (ULF), wetland diseased (WDF), and wetland healthy (WHF) rhizoplane samples, respectively (n = 3). Following quality trimming, 847,760 (ULF), 1,037,834 (WDF), and 539,722 (WHF) paired-end reads were retained. Subsequent read merging yielded 430,661, 522,889, and 274,139 ITS sequences for ULF, WDF, and WHF, respectively. Mean GC contents ranged from 48.5% to 54.3%. Reads clustered into 70 OTUs representing 48 fungal genera (**Table 1**), indicating adequate sequencing depth.

**Table 1:** Alpha-diversity metrics of cassava rhizoplane fungal communities revealed by ITS metabarcoding across upland and wetland habitats.

Metric	ULF (Upland)	WDF (Wetland diseased)	WHF (Wetland healthy)
Reads	847,760	1,037,834	539,722
GC (%)	50.7	48.5	54.3
Shannon	0.9	2.0	2.4
Observed richness	52.7	58.0	61.7
Chao1	57.0	60.7	63.4
Chao1 SE	2.3	3.2	5.0
ACE	59.1	61.6	65.1
ACE SE	3.1	3.5	3.8
Simpson	0.4	0.7	0.9
Inverse Simpson	1.8	4.3	7.0
Fisher	5.5	6.3	7.6

Alpha-diversity of cassava rhizoplane fungal communities based on ITS metabarcoding across upland (ULF), wetland diseased (WDF), and wetland healthy (WHF) habitats. Metrics include sequencing depth, GC content, diversity indices (Shannon, Simpson, Inverse Simpson, Fisher’s alpha), and richness estimators (Observed richness, Chao1, and ACE) with standard errors.

Fungal diversity varied markedly across habitats and plant health status. Upland samples exhibited the lowest diversity and evenness (Shannon 0.9; classical Simpson diversity index (D) 0.4), reflecting dominance by a few taxa. Diseased wetlands showed intermediate diversity (Shannon 2.0; classical Simpson diversity index (D) 0.7), whereas healthy wetlands had the highest diversity and evenness (Shannon 2.4; classical Simpson diversity index (D) 0.9). Richness estimators (Chao1, ACE) and Fisher’s alpha followed similar trends, indicating that wetland environments support higher fungal diversity, while disease is associated with community contraction.

### 3.2 Soil physicochemical drivers of mycobiome structure

Wetland soils were moderately acidic (pH 5.49) compared with uplands (pH 6.85) and were enriched in organic carbon, nitrogen, phosphorus, sulphur, boron, zinc, and copper (**Supplementary Table 1**). In contrast, uplands had higher potassium, calcium, iron, and manganese, with similar magnesium levels. These edaphic differences were reflected in mycobiome composition. Acidic, nutrient-rich wetland soils supported a higher abundance of yeast-associated taxa, whereas upland soils favoured filamentous fungi under better-aerated conditions. Together, soil chemistry and hydrological conditions shaped rhizoplane community structure and disease outcomes.

### 3.3 Fungal community composition across habitats

**Figure 1:**
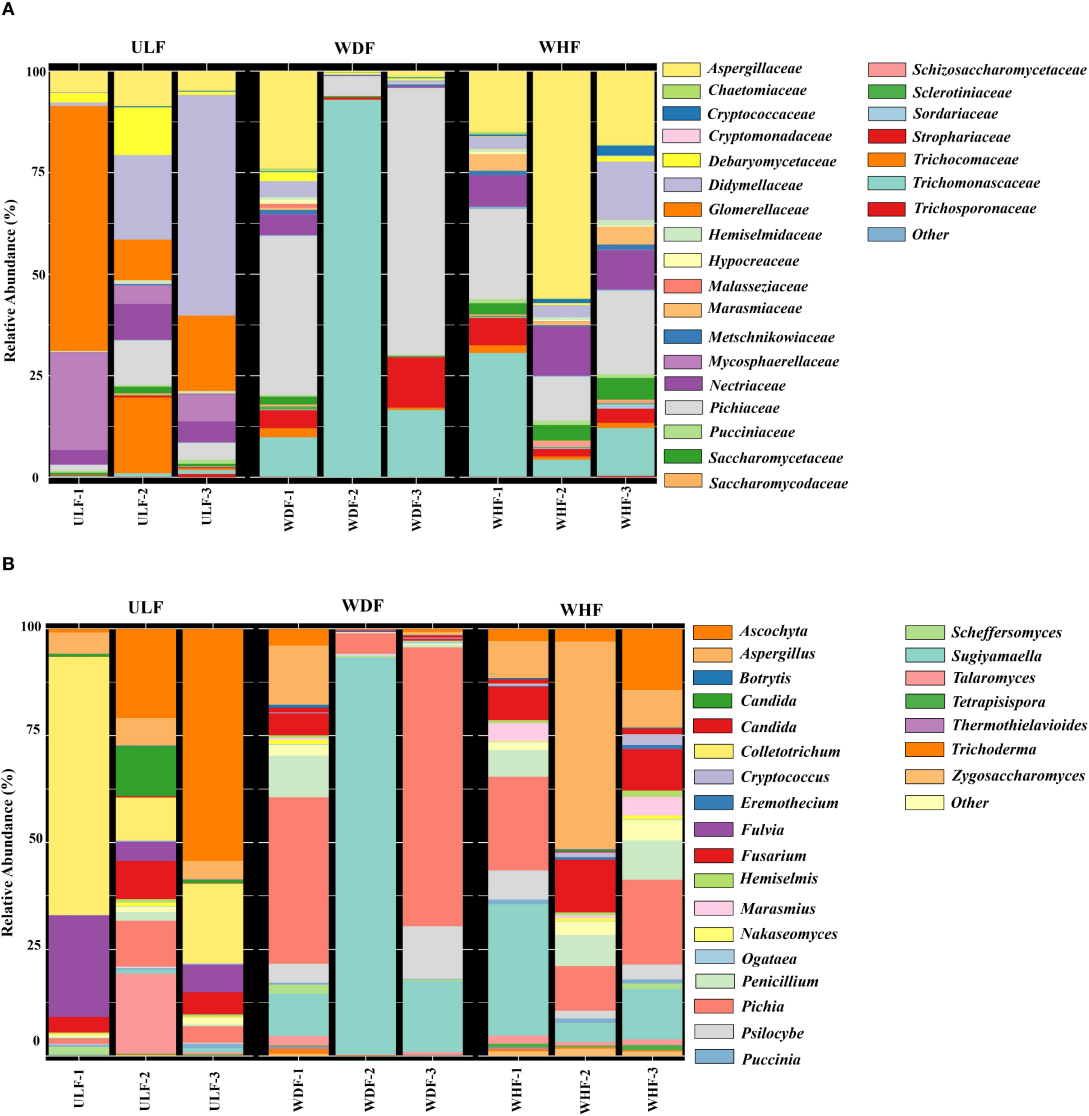
Fungal community composition of rhizoplane samples revealed by ITS amplicon sequencing: **(A)** family-level distribution and **(B)** genus-level distribution.

Fungal community composition differed clearly across habitats and plant health states (**Figure 1**). Ascomycota dominated all samples, followed by Basidiomycota (**Supplementary Figure 1**). At the class level, upland rhizoplanes were enriched in Dothideomycetes and Sordariomycetes, healthy wetlands in Eurotiomycetes and Agaricomycetes, and diseased wetlands showed a strong shift toward Saccharomycetes (**Supplementary Figure 2**).

At the order level, Glomerellales and Pleosporales dominated uplands, Eurotiales and Agaricales were prevalent in healthy wetlands, and Saccharomycetales were strongly enriched in diseased wetlands (**Supplementary Figure 3**). Family-level patterns reinforced these trends (**Figure 1A**, **Supplementary Table 2**), with uplands dominated by filamentous families (e.g., Glomerellaceae, Pleosporineae) and wetlands enriched in yeast-associated families, particularly Trichomonascaceae and Pichiaceae. Diseased wetlands showed a marked expansion of Trichomonascaceae (63.5% vs. 24.4% in healthy wetlands), whereas healthy wetlands retained greater family-level evenness.

Genus-level profiles showed similar patterns (**Figure 1B**, **Supplementary Table 3**). Uplands were dominated by filamentous genera such as *Colletotrichum*, *Ascochyta*, and *Fulvia*, whereas wetlands were dominated by yeasts including *Sugiyamaella* and *Pichia*. Diseased wetlands showed higher relative abundance increased dominance of these yeasts, while healthy wetlands maintained higher proportions of filamentous genera such as *Aspergillus*, *Fusarium*, *Penicillium*, and *Psilocybe*. Overall, diseased wetlands showed reduced evenness and dominance by a limited number of yeast taxa.

### 3.4 Alpha- and beta-diversity patterns

**Figure 2:**
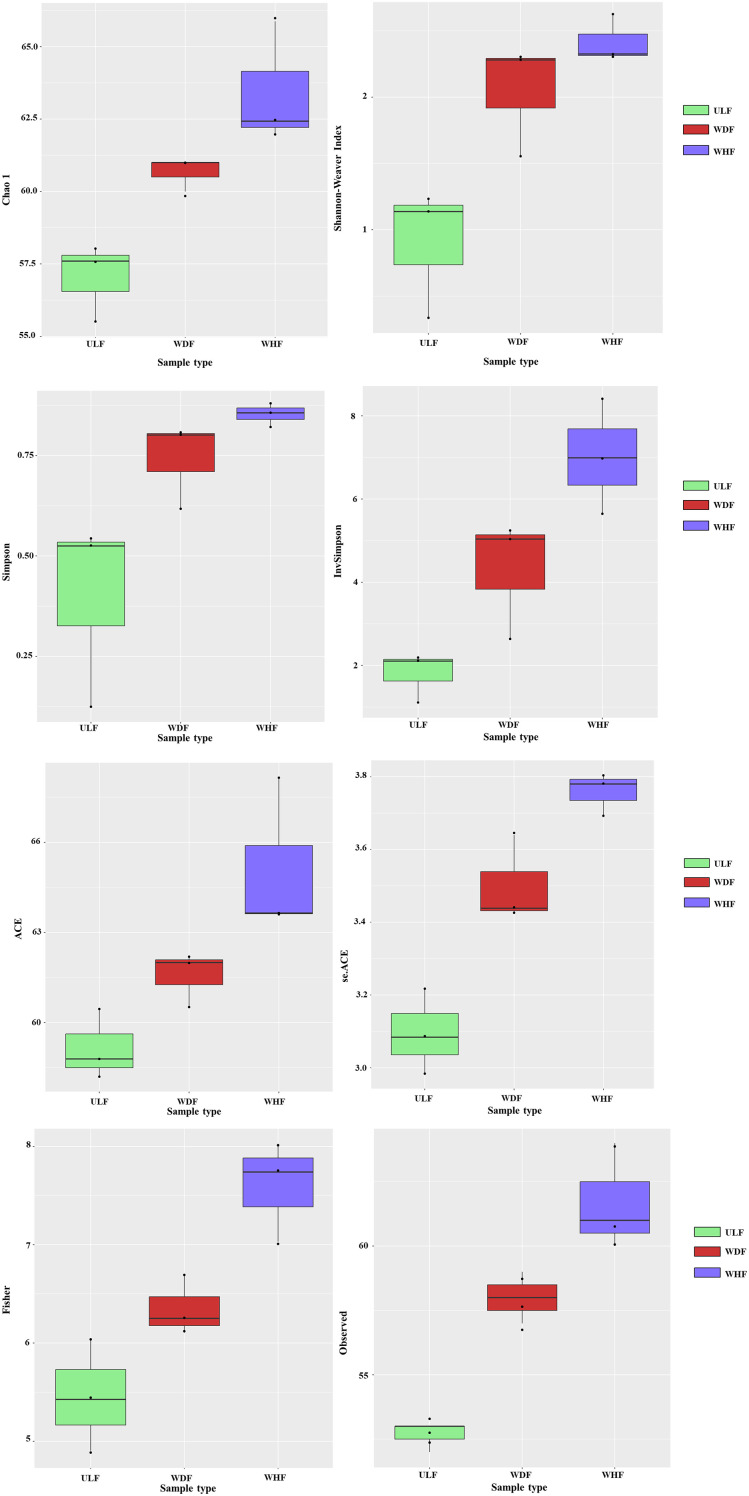
Alpha-diversity indices depicting richness and evenness of fungal communities in the cassava rhizoplane mycobiome. ULF, upland fungi; WDF, wetland diseased fungi; WHF, wetland healthy fungi.

**Figure 3:**
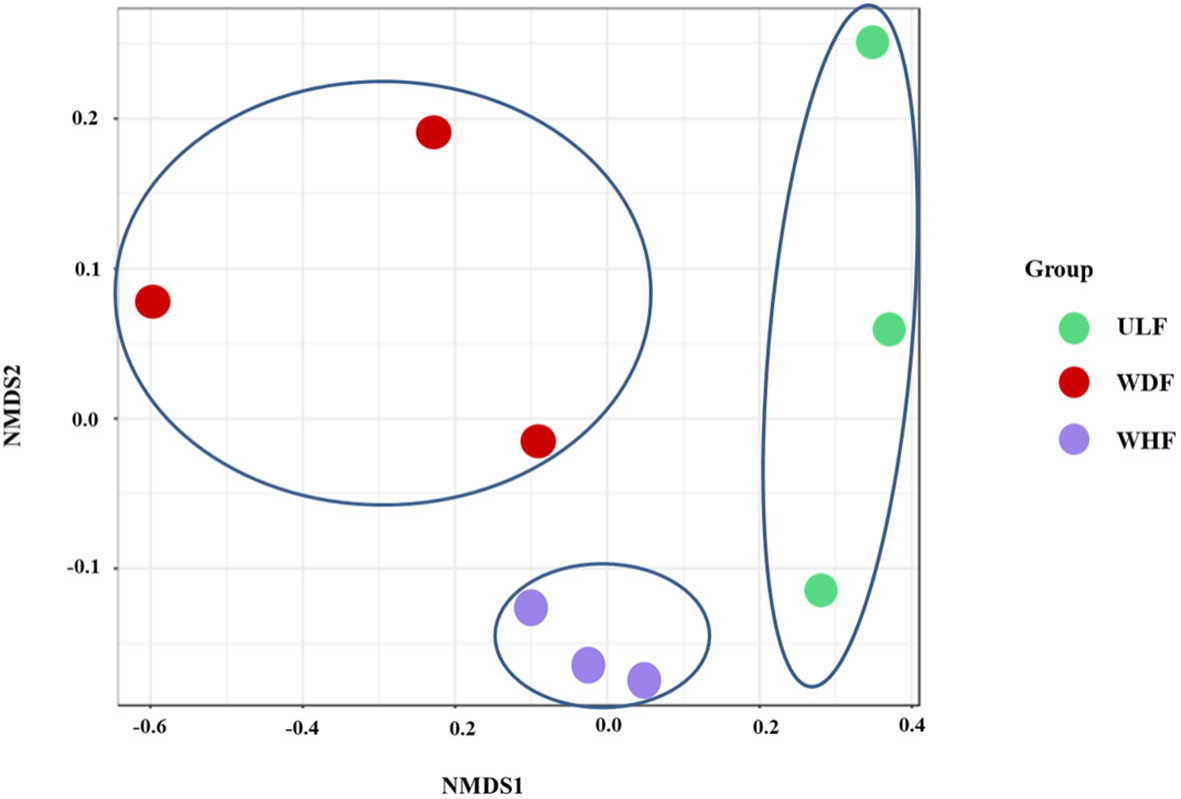
Beta-diversity of fungal communities in the cassava rhizoplane mycobiome based on NMDS ordination. Community differences among groups were supported by PERMANOVA (F = 2.937, R² = 0.4947, p = 0.012), with a low NMDS stress value (0.045) indicating a robust ordination. ULF, upland fungi; WDF, wetland diseased fungi; WHF, wetland healthy fungi.

Alpha-diversity indices increased from upland to diseased wetland and were highest in healthy wetlands (**Figure 2**). Healthy wetlands exhibited the greatest richness, diversity, and evenness, whereas uplands showed the lowest values. Diseased wetlands displayed reduced diversity compared to healthy wetlands. Beta-diversity analysis revealed clear separation by habitat and plant health (**Figure 3**). NMDS ordination showed distinct clustering of upland, healthy wetland, and diseased wetland samples. PERMANOVA confirmed significant differences among groups (R² = 0.495, p = 0.012), while PERMDISP indicated no significant differences in dispersion (p > 0.05), suggesting that variation was driven primarily by community composition rather than dispersion.

All major alpha-diversity indices (Observed richness, Chao1, ACE, Shannon, Simpson, InvSimpson, and Fisher’s alpha) remained significant after Benjamini–Hochberg FDR correction, supporting the robustness of these patterns. Upland rhizoplanes maintained a distinct, stable community associated with lower disease incidence, whereas healthy wetlands showed the greatest compositional consistency. Diseased wetlands exhibited higher dispersion, indicating increased community instability.

### 3.5 Sequencing depth assessment

Rarefaction curves approached saturation for all samples, confirming sufficient sequencing depth (**Supplementary Figure 4**). Healthy wetlands showed high richness with low variability, whereas diseased wetlands exhibited greater variability, consistent with community destabilization. Upland samples reached lower asymptotic richness, reflecting a reduced but distinct mycobiome.

### 3.6 Core and habitat-specific OTUs

**Figure 4:**
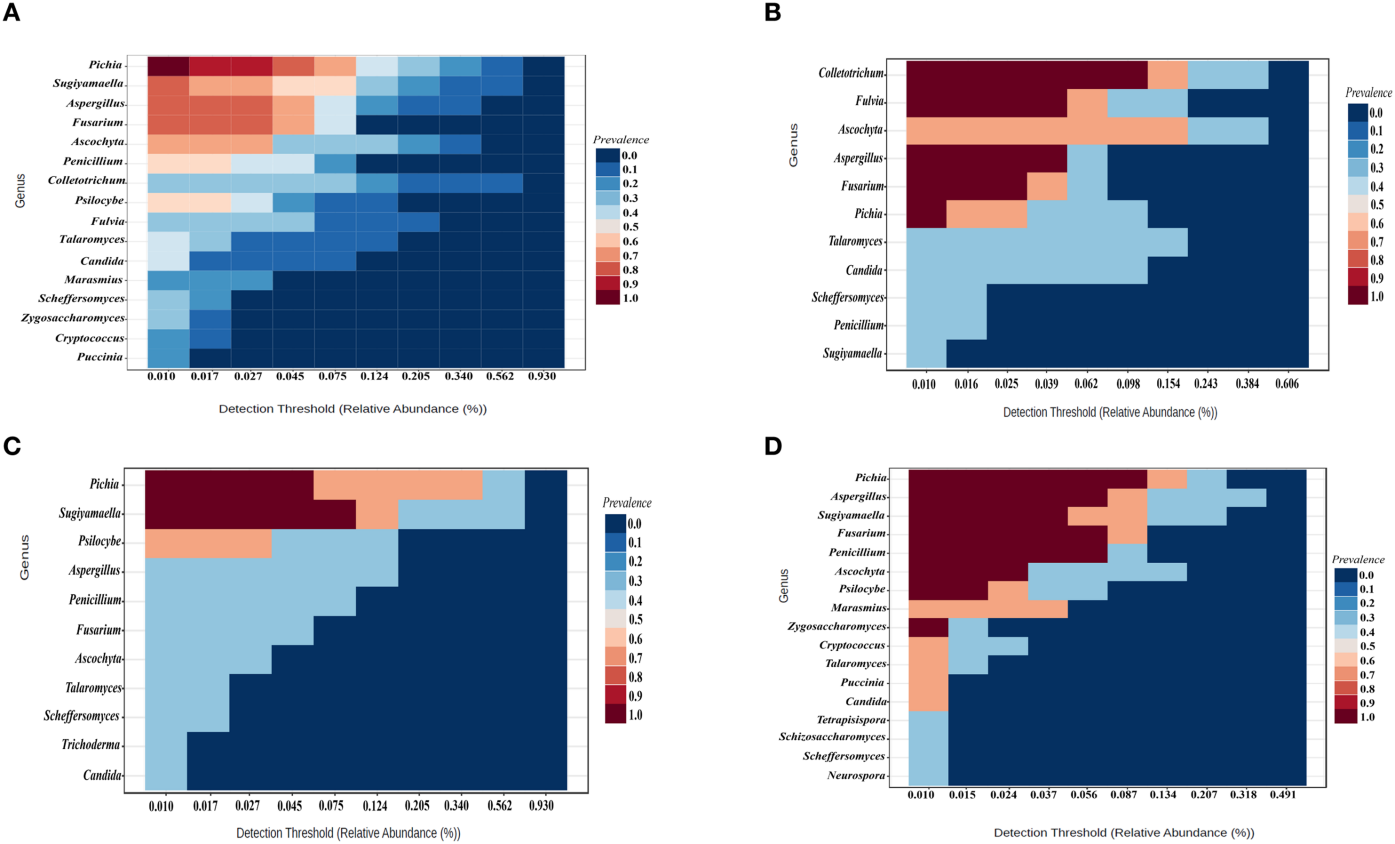
Core mycobiome of cassava rhizoplane; **(A)** rhizoplane of cassava representing upland, wetland diseased and wetland healthy agro ecosystems; **(B)** rhizoplane of cassava representing upland eubiosis state; **(C)** rhizoplane of cassava representing wetland dysbiosis state; **(D)** rhizoplane of cassava representing wetland eubiosis state.

Using a ≥70% prevalence threshold, the cassava rhizoplane core mycobiome was dominated by *Pichia* (100% prevalence), *Fusarium* (77.8%), and *Aspergillus* (77.8%) (**Figure 4**). An extended core included *Penicillium* and *Talaromyces*, both of which showed strong antagonistic activity against *Fusarium falciforme*. Habitat-specific differences were observed in relative abundance patterns. Uplands were dominated by filamentous taxa, healthy wetlands maintained a balanced yeast–filamentous core, and diseased wetlands showed a reduced core dominated by yeasts. OTU overlap analysis revealed a largely conserved community, with 60 OTUs shared across all habitats (**Supplementary Figure 5**). No habitat-exclusive OTUs were detected, indicating strong host filtering, with habitat and disease primarily influencing relative abundance rather than taxon presence.

### 3.7 Culture-based validation of metabarcoding results

Culture-dependent analysis recovered 83 fungal isolates representing 26 genera, broadly supporting metabarcoding results (**Table 2**, **Supplementary Figure 6**). Core taxa identified by sequencing, including *Aspergillus*, *Penicillium*, and *Talaromyces*, were consistently isolated. Upland rhizoplanes showed a stable, filamentous-dominated culturable community. Diseased wetlands exhibited reduced representation of beneficial filamentous taxa, whereas healthy wetlands displayed the highest culturable diversity. These findings validate the sequencing-derived core mycobiome and highlight disease-associated dysbiosis.

**Table 2:** Cultivable fungal communities associated with cassava rhizoplane habitats and their core microbiome status.

Habitat	Fungal genus (No.)	*Fungal species (Isolates)	**GenBank accession No.	***Core microbiome
Upland	*Aspergillus* (21)	*A. aculeatus, A. allahabadii, A. citrinoterreus*, *A. corrugatus, A. flavus, A. fumigatus, A. niger, A. nomiae, A. ochraceus, A. oryzae, A. pseudoterreus, Aspergillus* sp., *A. tamarii, A. terreus, A. urmiensis*	PX651284–PX651310	**Yes**
	*Cyberlindnera* (1)	*Cyberlindnera suaveolens*	PX651317	**Yes**
	*Meyerozyma* (1)	*Meyerozyma* sp.	PX651318	**Yes**
	*Penicillium* (5)	*P. chrysogenum, P. citrinum, P. menonorum*, *P. pimiteouiense, P. pinophilum*	PX651307, PX651293, PX651311, PX651288, PX651286	**Yes**
	*Talaromyces* (2)	*T. pinophilus, T. verruculosus*	PX653175, PX651303	**Yes**
	*Fusarium* (1)	*Fusarium oxysporum*	PX651313	**Yes**
	*Emericella* (1)	*Emericella parvathecia*	PX651292	No
	*Glomerella* (1)	*Glomerella cingulate*	PX651316	No
	*Hypoxylon* (1)	*Hypoxylon fragiforme*	PX651290	No
	*Mucor* (1)	*Mucor irregularis*	PX651312	No
	*Pseudolagarobasidium* (1)	*P. acaciicola*	PX651294	No
	*Rhizopycnis* (1)	*Rhizopycnis vagum*	PX651314	No
	*Trichocladium* (1)	*Trichocladium amorphum*	PX651315	No
Wetland Diseased	*Aspergillus* (1)	*Aspergillus aculeatus*	PX631811	**Yes**
	*Cyberlindnera* (1)	*Cyberlindnera saturnus*	PX631834	**Yes**
	*Fusarium* (2)	*F. incarnatum, F. solani*	PX631832, PX631831	**Yes**
	*Penicillium* (2)	*P. oxalicum, P. raperi*	PX631812, PX631813	**Yes**
	*Talaromyces* (1)	*Talaromyces* sp.	PX631829	**Yes**
	*Trichoderma* (2)	*T. asperellum, Trichoderma* sp.	PX631825, PX631815	**Yes**
	*Meyerozyma* (1)	*Meyerozyma guilliermondii*	PX631833	**Yes**
	*Cunninghamella* (1)	*Cunninghamella* sp.	PX631814	No
	*Curvularia* (1)	*Curvularia lunata*	PX631830	No
	*Mucor* (1)	*Mucor* sp.	PX631827	No
	*Nectria* (1)	*Nectria mauritiicola*	PX631828	No
	*Stemphylium* (1)	*Stemphylium vesicarium*	PX631826	No
Wetland Healthy	*Aspergillus* (6)	*A. fumigatus, A. nomiae, Aspergillus* sp., *A. tamari, A. versicolor, A. welwitschiae*	PX651269, PX651258, PX651270, PX651259, PX651274, PX651253	**Yes**
	*Fusarium* (1)	*Fusarium solani*	PX651272	**Yes**
	*Meyerozyma* (1)	*Meyerozyma guilliermondii*	PX651279	**Yes**
	*Penicillium* (7)	*P. janthinellum, P. oxalicum, P. pinophilum, P. raperi, Penicillium* sp.	PX651263–PX651276	**Yes**
	*Williopsis* (1)	*Williopsis saturnus*	PX651278	**Yes**
	*Trichoderma* (2)	*T. asperellum, Trichoderma* sp.	PX651260, PX651250	**Yes**
	*Absidia* (1)	*Absidia cuneospora*	PX651273	No
	*Chaetomium* (2)	*Chaetomium* sp.	PX651266, PX651267	No
	*Cunninghamella* (2)	*C. bertholletiae*	PX651261, PX651265	No
	*Gongronella* (1)	*Gongronella butleri*	PX651277	No
	*Humicola* (1)	*Humicola olivacea*	PX651264	No
	*Lasiodiplodia* (1)	*Lasiodiplodia theobromae*	PX651262	No
	*Macrophomina* (1)	*Macrophomina phaseolina*	PX651255	No
	*Mucor* (2)	*M. ellipsoideus, M. irregularis*	PX651271, PX651254	No
	*Nigrospora* (1)	*Nigrospora* sp.	PX651268	No

Figures in parentheses indicate the number of isolates belonging to each fungal genus; *Sequences generated by ITS sequencing technology were used to identify the fungi at species level; **All sequences are submitted to GenBank https://www.ncbi.nlm.nih.gov/nucleotide/; ***The isolated genera were compared with the core microbiome data determined through metabarcoding. Bold text indicates fungal taxa identified as members of the core microbiome.

### 3.8 Antagonistic activity against *Fusarium falciforme*

**Figure 5:**
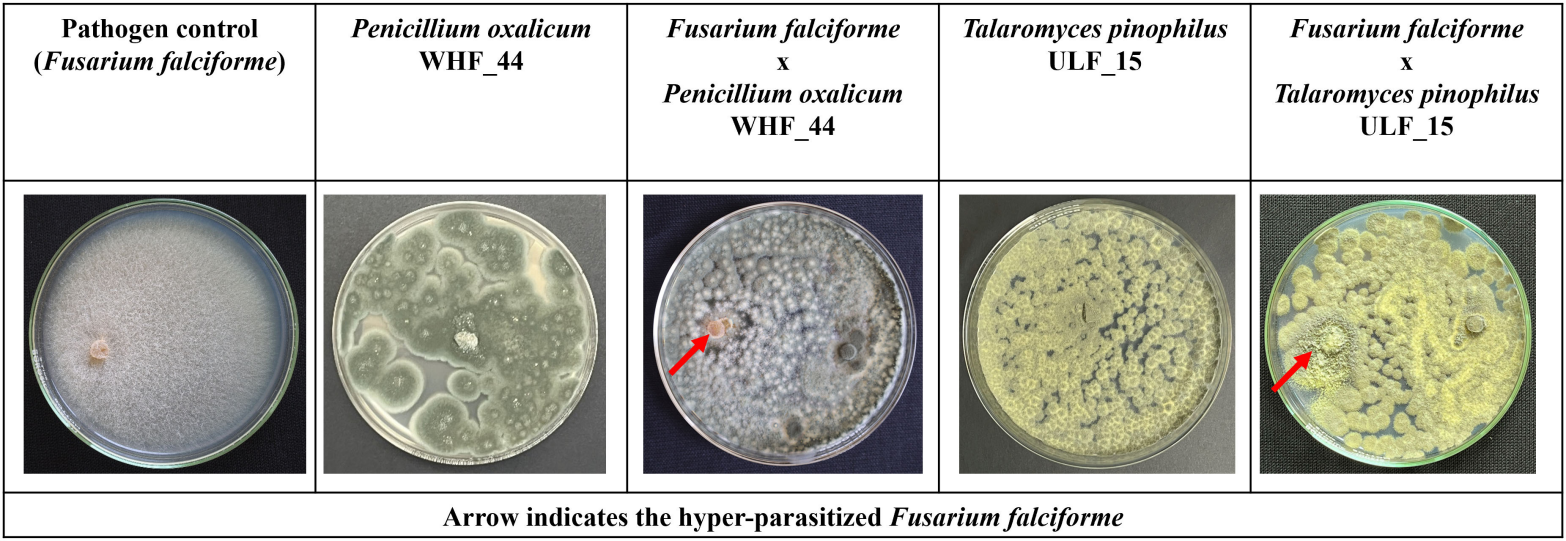
Dual culture assay demonstrating the antagonistic potential of rhizoplane fungal isolates against *Fusarium falciforme*.

Fungal isolates showed a wide range of antagonistic activity against *F. falciforme*, from weak (<30%) to very strong (>90%) inhibition (p ≤ 0.05; **Table 3**). Upland isolates displayed broad-spectrum activity, led by *Talaromyces pinophilus* (90.0%) and *Aspergillus aculeatus* (88.2%). Diseased wetland isolates generally showed reduced antagonistic potential, whereas healthy wetland isolates exhibited the strongest and most consistent inhibition, with *Penicillium oxalicum* achieving up to 92.6% suppression (**Supplementary Figures 7**–**9**). Core genera (*Penicillium*, *Aspergillus*, *Talaromyces*, *Trichoderma*) consistently inhibited the pathogen, whereas yeast taxa showed weak activity. These results identify *P. oxalicum* and *T. pinophilus* as key biocontrol candidates (**Figure 5**).

**Table 3:** *In vitro* antifungal activity of rhizoplane fungal isolates from upland (ULF), wetland diseased (WDF), and wetland healthy (WHF) habitats against *Fusarium falciforme*.

Upland			
Isolate	PI	Isolate	PI
*Talaromyces pinophilus*ULF-15	90.00 ± 1.11a	*Aspergillus ochraceus*-2ULF-9	55.56 ± 2.94g
*Aspergillus aculeatus*ULF-4	88.15 ± 0.64ab	*Pseudolagarobasidium acaciicola*ULF-14	55.19 ± 2.57g
*Aspergillus terreus*ULF-17	84.44 ± 1.11bc	*Mucor irregularis*ULF-64	54.44 ± 1.92g
*Aspergillus niger*ULF-3	82.96 ± 1.70cd	*Hypoxylon fragiforme*ULF-10	54.44 ± 0.00g
*Aspergillus flavus*-3ULF-23	82.59 ± 2.57cd	*Aspergillus* sp.-1ULF-20	53.33 ± 1.92g
*Aspergillus flavus*-2ULF-11	81.85 ± 3.39cd	*Talaromyces verruculosus*ULF-24	52.96 ± 3.57g
*Aspergillus allahabadii*-1ULF-22	81.48 ± 1.28cd	*Aspergillus pseudoterreus*ULF-18	52.59 ± 3.39gh
*Aspergillus allahabadii*-2ULF-19	81.11 ± 2.94cd	*Aspergillus corrugatus*ULF-31	48.89 ± 2.94hi
*Aspergillus fumigatus*-2ULF-21	80.00 ± 2.94d	*Glomerella cingulata*ULF-79	47.04 ± 1.28ij
*Aspergillus nomiae*ULF-7	75.56 ± 2.94e	*Trichocladium amorphum*ULF-70	47.04 ± 1.28ij
*Aspergillus fumigatus*-1ULF-26	72.96 ± 3.39e	*Fusarium oxysporum*ULF-68	45.56 ± 1.11ij
*Aspergillus citrinoterreus*ULF-5	72.59 ± 2.80e	*Aspergillus urmiensis*ULF-33	43.33 ± 1.92jk
*Emericella parvathecia*ULF-12	65.56 ± 1.92f	*Aspergillus* sp.-2ULF-32	40.37 ± 1.70kl
*Aspergillus oryzae*ULF-1	63.70 ± 3.39f	*Penicillium menonorum*ULF-63	39.26 ± 1.28lm
*Aspergillus tamarii*ULF-25	63.33 ± 3.33f	*Penicillium pimiteouiense*ULF-8	38.89 ± 0.00lm
*Penicillium pinophilum*ULF-6	62.59 ± 1.70f	*Rhizopycnis vagum*ULF-69	35.93 ± 2.80m
*Penicillium citrinum*ULF-13	62.22 ± 1.11f	*Meyerozyma* sp.ULF-5Y	31.85 ± 0.64n
*Aspergillus flavus*-1ULF-16	55.56 ± 2.22g	*Penicillium chrysogenum*ULF-30	30.74 ± 2.80n
*Aspergillus ochraceus*-1ULF-29	55.56 ± 2.22g	*Cyberlindnera suaveolens*ULF-1Y	24.81 ± 1.70o
Wetland diseased			
*Aspergillus aculeatus*WDF1-38	85.19 ± 1.70a	*Talaromyces* sp. WDF10-28	50.00 ± 1.11f
*Penicillium oxalicum*WDF2-37	77.04 ± 2.31b	*Fusarium solani*WDF12-71	47.78 ± 0.00fg
*Penicillium raperi*WDF3-34	76.30 ± 1.70b	*Curvularia lunata*WDF11-36	45.93 ± 3.57gh
*Trichoderma asperellum*WDF6-40	66.30 ± 0.64c	*Fusarium incarnatum*WDF13-78	44.44 ± 0.00hi
*Trichoderma sp*WDF5-41	64.44 ± 1.11cd	*Nectria mauritiicola*WDF9-39	42.59 ± 0.64i
*Stemphylium vesicarium*WDF7-42	63.33 ± 1.11d	*Meyerozyma guilliermondii*WDF14-6Y	32.22 ± 1.92j
*Cunninghamella* sp. WDF4-62	62.96 ± 1.70d	*Cyberlindnera saturnus*WDF15-2Y	25.56 ± 3.33k
*Mucor* sp. WDF8-35	56.67 ± 1.11e		
Wetland healthy			
*Penicillium oxalicum*-1WHF-44	92.59 ± 1.05^a^	*Mucor ellipsoideus*WHF-67	55.19 ± 1.39^hi^
*Penicillium oxalicum*-2WHF-50	89.63 ± 0.52^a^	*Aspergillus tamarii*WHF-52	54.81 ± 0.52^ij^
*Aspergillus welwitschiae*WHF-46	84.44 ± 2.72^b^	*Trichoderma asperellum*WHF-53	54.07 ± 1.39^ij^
*Aspergillus fumigatus*WHF-65	82.59 ± 2.10^b^	*Nigrospora* sp.WHF-61	51.85 ± 1.39^jk^
*Cunninghamella bertholletiae*-1WHF-58	77.41 ± 0.52^c^	*Chaetomium* sp.-2WHF-59	50.74 ± 1.05^kl^
*Aspergillus nomiae*WHF-51	76.30 ± 1.39^c^	*Absidia cuneospora*WHF-72	50.37 ± 0.52^kl^
*Penicillium janthinellum*WHF-56	74.44 ± 2.40^cd^	*Fusarium solani*WHF-71	49.63± 0.52^klm^
*Cunninghamella bertholletiae*-2 WHF-54	72.59 ± 1.05^de^	*Chaetomium* sp.*-1*WHF-60	47.78 ± 2.40^lm^
*Penicillium pinophilum*WHF-49	72.22 ± 0.91^de^	*Gongronella butleri*WHF-77	46.67 ± 0.91^m^
*Aspergillus* sp.WHF-66	71.85 ± 2.92^de^	*Humicola olivacea*WHF-57	34.81 ± 2.28^n^
*Lasiodiplodia theobromae*WHF-55	71.11 ± 0.91^e^	*Aspergillus versicolor*WHF-73	32.22 ± 0.91^no^
*Trichoderma* sp.WHF-43	65.56 ± 0.91^f^	*Meyerozyma guilliermondii*WHF-7Y	31.11 ± 1.81^o^
*Penicillium raperi*WHF-45	60.00 ± 1.81^g^	*Penicillium* sp.-2WHF-75	27.78 ± 0.91^p^
*Mucor irregularis*WHF-47	58.89 ± 2.40^g^	*Penicillium* sp.-1WHF-74	26.67 ± 1.81^p^
*Macrophomina phaseolina*WHF-48	58.15 ± 1.39^gh^	*Williopsis saturnus*WHF-3Y	25.56 ± 2.40^p^

Values represent percent inhibition (PI, mean ± SD; n = 3). Different lowercase letters within each habitat indicate significant differences among isolates according to ANOVA followed by *post hoc* comparison at p ≤ 0.05.

### 3.9 Greenhouse validation of antagonistic core fungal isolates

**Figure 6:**
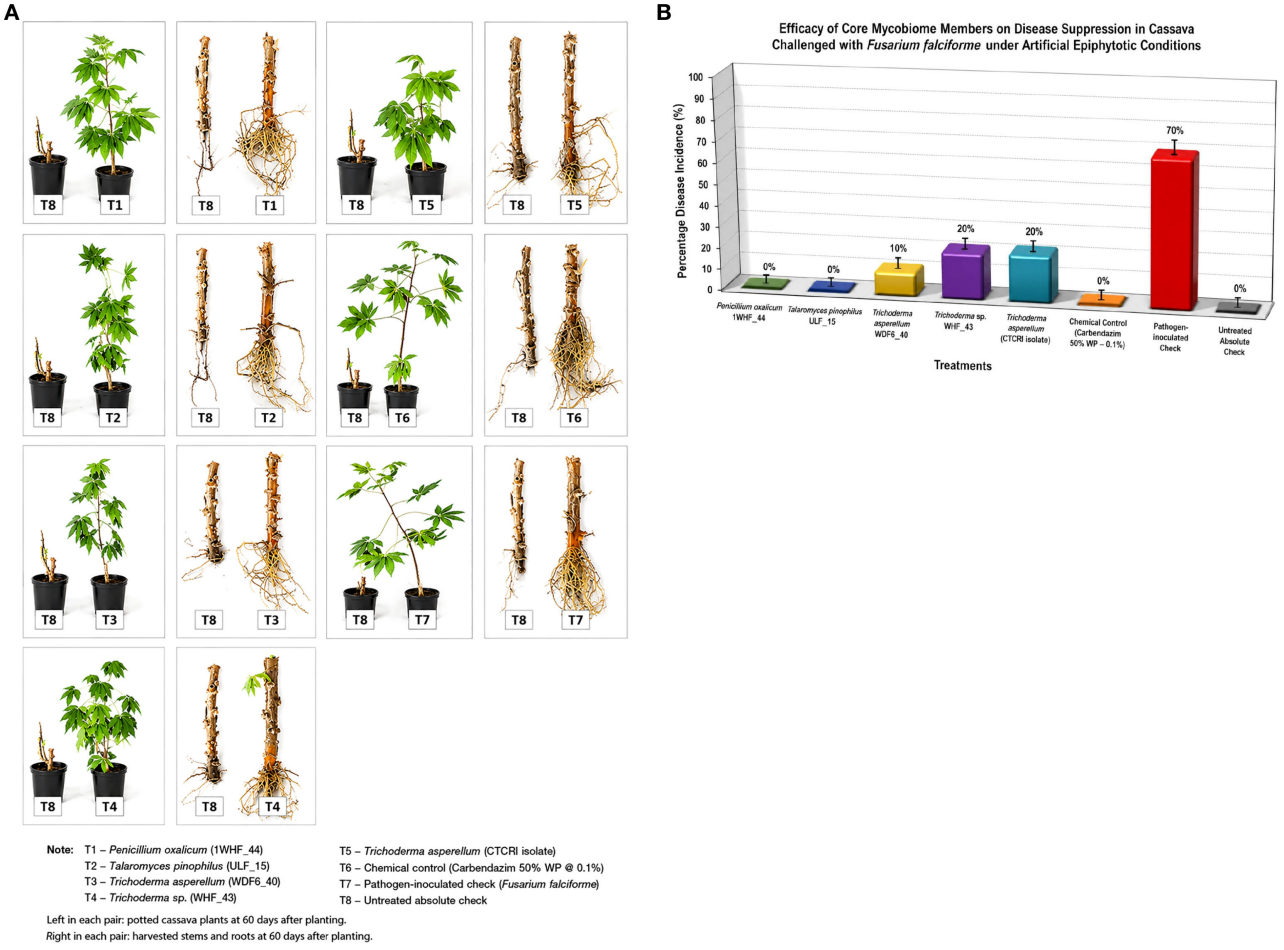
Efficacy of core mycobiome members in suppressing cassava stem and root rot caused by *Fusarium falciforme* under artificial epiphytotic conditions. **(A)** Comparative plant growth response of cassava under different treatments at 60 days after planting, and **(B)** corresponding disease incidence percentage.

Significant treatment effects were observed for disease incidence and all growth parameters (p < 0.01), indicating strong responses under greenhouse conditions. The pathogen-inoculated control showed severe disease (70% incidence) and substantial reductions in plant growth and root development compared to all other treatments (**Figure 6b**). *Penicillium oxalicum* 1WHF_44 showed the highest efficacy, completely suppressing disease (0% incidence), comparable to the uninoculated control and chemical treatment (carbendazim 0.1%). It also recorded the highest values for all growth parameters (**Supplementary Table 4**). *Talaromyces pinophilus* ULF_15 similarly achieved complete disease suppression and performed on par with *P. oxalicum* and the chemical control. Rhizoplane-derived *Trichoderma* isolates significantly reduced disease and improved plant growth but were less effective than *P. oxalicum* and *T. pinophilus*. Their performance was comparable to the recommended CTCRI *T. asperellum* isolate for several traits. Collectively, greenhouse validation confirmed that core antagonistic fungi identified through microbiome analysis are both ecologically persistent and functionally effective. High Cohen’s *F* values across traits further indicated strong treatment effects and biological relevance.

## 4 Discussion

### 4.1 Rhizoplane mycobiome structure and disease-associated dysbiosis

This study provides a comparative analysis of the cassava rhizoplane mycobiome across disease-endemic wetland and non-endemic upland systems, highlighting the rhizoplane as a critical microbial interface influencing plant health. While previous work has largely focused on the rhizosphere, our results demonstrate that the rhizoplane-acting as the immediate microbial barrier at the root surface-plays a key role in determining disease outcomes. Across all samples, Ascomycota was the dominant phylum, consistent with earlier reports on cassava-associated fungal communities (Cai et al., 2021; Liao et al., 2026). However, clear differences emerged at lower taxonomic levels, reflecting strong habitat and health-dependent structuring of the mycobiome. Healthy plants maintained relatively balanced and structured fungal communities, whereas diseased wetland plants showed pronounced compositional shifts, including enrichment of Trichomonascaceae and reduced representation of Pichiaceae and Aspergillaceae. Such changes are indicative of microbial dysbiosis and are consistent with the concept of pathobiome formation (Jakuschkin et al., 2016; Thomas et al., 2025). The decline of genera such as *Aspergillus* and *Penicillium* may suggest a potential disruption of beneficial fungal interactions and functions that contribute to rhizosphere stability and pathogen suppression, thereby enabling opportunistic fungi to proliferate (Mendes et al., 2013; Gao et al., 2025). Similar patterns have been reported in other crop systems, including banana and sweet potato, where disease development is associated with reduced microbial diversity and altered community structure (Kaushal et al., 2020; Wang et al., 2023). Together, these findings support the view that cassava stem and root rot is linked to broader ecological disruption rather than the presence of a single pathogen.

### 4.2 Habitat filtering and edaphic drivers of mycobiome assembly

Habitat strongly influenced fungal community composition. Wetland rhizoplanes were dominated by yeast-associated taxa, particularly Trichomonascaceae, whereas upland systems supported filamentous fungi such as members of Glomerellaceae, Pleosporineae, and Mycosphaerellaceae. Similar habitat-driven microbial differentiation has been reported in other terrestrial ecosystems (Li et al., 2018). The detection of phytopathogenic genera such as *Colletotrichum*, *Ascochyta*, and *Fulvia* in upland soils indicates that non-endemic systems can harbor latent pathogen reservoirs. However, the absence of disease symptoms suggests that microbial community structure and soil conditions regulate pathogen activity rather than eliminate pathogens (Gao et al., 2019). In this context, the rhizoplane functions as a microbial filter influencing plant health (Compant et al., 2019). Healthy wetland plants maintained communities enriched in *Sugiyamaella*, *Pichia*, and *Aspergillus*, whereas diseased plants showed reduced diversity and altered composition, consistent with studies showing that wetland environmental conditions and degradation significantly influence fungal community composition, diversity, and stability (Berg et al., 2020; Yan et al., 2024).

Soil physicochemical properties were closely linked to these patterns. Wetland soils were more acidic and enriched in nitrogen and phosphorus but relatively deficient in potassium and calcium, conditions previously associated with increased *Fusarium* prevalence and altered microbial networks (Cruz et al., 2019; Orr et al., 2022; Li et al., 2023; Jing et al., 2025; Lem et al., 2025). Prolonged soil saturation may further reduce microbial network complexity and keystone taxa, thereby destabilizing microbial interactions and ecosystem resilience (Li et al., 2018; Maurice et al., 2024). Fertilization and nutrient availability are also known to reshape cassava-associated fungal communities (Cai et al., 2021; Liu et al., 2025), while elevated sulphur under anaerobic conditions may promote disease-favouring metabolic pathways (Dong et al., 2024). In contrast, upland soils with near-neutral pH, balanced fertility, and better aeration supported more stable microbial assemblages and lower disease incidence (Jeeva et al., 2020). Given the cross-sectional nature of this study, these relationships should be interpreted as correlative. Disease-associated shifts may arise both from environmental stress and pathogen-induced changes in root physiology, leading to feedback effects on microbial community structure. Overall, cassava stem and root rot appear to result from interactions among pathogen presence, soil acidity, nutrient imbalance, and hydrological stress rather than pathogen occurrence alone. Furthermore, we acknowledge that a limitation of this field-based study is the absence of a standardised quantitative disease severity index (e.g., a 0–5 disease rating scale) during sampling. Although plants were categorized based on the presence or absence of characteristic stem and root rot symptoms, incorporating a quantitative severity index in future studies would provide deeper insights into progressive microbiome shifts across different stages of disease development.

### 4.3 Microbial diversity and functional stability in disease suppression

A strong association was observed between microbial diversity and disease suppression (Berg et al., 2017). Healthy wetland rhizoplanes exhibited higher alpha diversity and lower variability, whereas diseased samples showed reduced diversity and increased dispersion. This pattern aligns with findings from other crop systems, where disease is associated with microbial dysbiosis and destabilization of beneficial communities (Darriaut et al., 2024; Wang et al., 2025). Higher diversity in healthy systems supports the concept that diverse microbial communities can suppress pathogen establishment through competition, nutrient blocking, and niche exclusion (Li et al., 2021, 2024). The enrichment of antagonistic fungi in healthy wetlands further suggests that environmental conditions influence not only community composition but also functional potential. Wetland environments, characterized by fluctuating redox conditions, may select for metabolically versatile fungi with enhanced stress tolerance and antagonistic capacity (Pezeshki and DeLaune, 2012; Liang et al., 2023).

Maintaining microbial diversity is essential for ecosystem stability, as diverse microbial communities enhance functional resilience, while beneficial taxa strengthen resistance to both biotic and abiotic disturbances (Raaijmakers et al., 2009; Gupta et al., 2022; Samadli, 2026). Reduced diversity in diseased wetlands was associated with increased disease incidence and destabilized fungal networks, consistent with patterns reported in multiple crop systems affected by *Fusarium* (Kaushal et al., 2020; Zhang et al., 2022; Wang et al., 2023; Wu et al., 2025; Tao et al., 2025). Beta diversity patterns further supported this interpretation, with healthy samples forming tighter clusters and diseased samples showing greater dispersion, indicative of increased community variability (Hooks and O’Malley, 2017).

### 4.4 Core mycobiome and functional validation of antagonistic taxa

The identification of a conserved rhizoplane core microbiome across habitats suggests strong host-mediated microbial selection, consistent with recent evidence that host-driven microbiome assembly enriches beneficial taxa and enhances disease resistance (Wu et al., 2026; Zhang et al., 2021). Core taxa, including *Pichia*, *Aspergillus*, *Sugiyamaella*, *Fusarium*, and *Ascochyta*, persisted across environmental conditions. The consistent presence of *Pichia* may reflect functional roles linked to metabolic versatility and antagonistic interactions (Kowalska et al., 2022; Elkhairy et al., 2023), while the persistence of *Fusarium* highlights its ecological plasticity and dual saprotrophic–pathogenic lifestyle (Ma et al., 2013; Armer et al., 2024). A key finding of this study is the identification of an extended functional core comprising *Penicillium oxalicum* and *Talaromyces pinophilus*. These taxa were not only consistently detected but also exhibited strong antagonistic activity against *Fusarium falciforme*, suggesting their role in a microbiome-mediated barrier effect at the rhizoplane. Accessory taxa such as *Candida*, *Zygosaccharomyces*, and *Colletotrichum* showed habitat-specific distributions, indicating a hierarchical organization with a stable core and a flexible accessory community. Similar patterns have been reported in other crop systems (Kumar et al., 2023; Guo et al., 2024; Ma et al., 2025). Culture-dependent analysis corroborated these findings, yielding 83 fungal isolates representing 26 genera, including *Aspergillus*, *Penicillium*, *Talaromyces*, and *Trichoderma*. These genera are well known for their production of antifungal metabolites and their mycoparasitic activity (Harman et al., 2004; Luo et al., 2021; Gangaraj et al., 2023; Dastogeer et al., 2026). Dual culture assays confirmed strong antagonistic activity, particularly for *P. oxalicum* and *T. pinophilus*. Diseased wetland samples yielded fewer effective antagonists, suggesting reduced persistence of beneficial fungi under pathogen-dominated conditions (Wei et al., 2019). In upland systems, *T. pinophilus* showed strong inhibition, consistent with previous reports of its biocontrol potential (Patel et al., 2021; Bennacer et al., 2025). The consistent detection of *Fusarium* in both healthy and diseased rhizoplane samples suggests that it may be a conditionally pathogenic core member of the cassava rhizoplane mycobiome. Accordingly, the mere presence of *Fusarium* does not necessarily indicate active disease; rather, its transition from a latent or commensal state to a pathogenic state is likely influenced by microbial community structure, inter-microbial interactions, environmental conditions, and host susceptibility.

### 4.5 Greenhouse validation and implications for disease management

Greenhouse validation confirmed the functional relevance of the identified antagonistic taxa. Both *P. oxalicum* and *T. pinophilus* completely suppressed disease incidence and significantly improved plant growth, performing comparably to chemical control. These findings are consistent with earlier reports demonstrating antifungal and plant growth–promoting activities of *Penicillium* and *Talaromyces* species (De Cal et al., 2009; Patel et al., 2021; Bennacer et al., 2025; Hossain et al., 2025). Although rhizoplane-derived *Trichoderma* isolates were somewhat less effective, they significantly reduced disease incidence and performed comparably to the recommended reference strain, suggesting that native isolates may be well adapted to cassava-associated environments.

The strong agreement between microbiome profiling, *in vitro* antagonism, and greenhouse validation highlights the ecological and functional significance of the identified taxa. These findings support a model in which cassava stem and root rot is driven not only by pathogen presence but also by environmentally mediated disruption of the rhizoplane microbiome. Integrated management strategies focusing on soil pH correction, balanced nutrient management, improved drainage, and conservation of beneficial microbes-along with targeted application of biocontrol agents such as *P. oxalicum* and *T. pinophilus*-offer a promising, climate-resilient approach to disease management. However, this study is limited to a single region and growing season. Future research should assess the temporal stability of these patterns across environments, seasons, and cassava genotypes, and further elucidate the mechanisms underlying microbial interactions and pathogen suppression to enable field-level application.

## 5 Conclusion

Cassava stem and root rot reflects a broader ecological shift in the rhizoplane, where the loss of functional balance-rather than just a reduction in species richness-drives disease development. Our findings indicate that disease suppression depends on a stable and functionally coordinated fungal community, in which core and extended members such as *Pichia*, *Penicillium*, *Aspergillus*, and *Talaromyces* work together to limit the establishment of *Fusarium falciforme*. This protective effect breaks down in diseased wetlands, where acidic, nutrient-imbalanced soils and prolonged waterlogging disrupt microbial interactions and favour opportunistic fungi. Interestingly, upland systems, despite having lower overall diversity, maintained more stable fungal communities and showed lower disease incidence, suggesting that community stability and functional capacity are more important than diversity alone. The strong agreement between sequencing data, culture-based assays, and greenhouse validation—where *Penicillium oxalicum* and *Talaromyces pinophilus* completely suppressed disease and improved plant growth—highlights the key role of these antagonists in maintaining rhizoplane health. Taken together, the study supports a view of cassava disease as an outcome of microbiome imbalance shaped by soil and environmental conditions, and points to the combined use of beneficial microbes and soil health management as a practical and sustainable strategy for disease control in flood-prone systems.

## Data Availability

The original contributions presented in the study are publicly available. This data can be found here: NCBI, accession: PRJNA1391667.

## Supplementary material

Supplementary Figure 1 Distribution and abundance of cassava rhizoplane mycobiome at the phylum level.

Supplementary Figure 2 Distribution and abundance of cassava rhizoplane mycobiome at the class level.

Supplementary Figure 3 Distribution and abundance of cassava rhizoplane mycobiome at the order level.

Supplementary Figure 4 The rarefaction curve represents the richness of species among the extracted microbiome samples.

Supplementary Figure 5 Shared taxa of the three rhizoplane mycobiome sample groups.

Supplementary Figure 6 Fungal isolates obtained from contrasting cassava cultivation practices.

Supplementary Figure 7 Bio-efficacy of wetland diseased rhizoplane mycobiome isolates.

Supplementary Figure 8 Bio-efficacy of wetland healthy rhizoplane mycobiome isolates.

Supplementary Figure 9 Bio-efficacy of upland rhizoplane mycobiome isolates.

## References

[B1] Alonso Chavez V. Milne A. E. Van den Bosch F. Pita J. McQuaid C. F. (2022). Modelling cassava production and pest management under biotic and abiotic constraints. Plant Mol. Biol. 109, 325–349. doi: 10.1007/s11103-021-01170-8 34313932 PMC9163018

[B2] Armer V. J. Kroll E. Darino M. Smith D. P. Urban M. Hammond-Kosack K. E. (2024). Navigating the Fusarium species complex: Host-range plasticity and genome variations. Fungal Biol. 128, 2439–2459. doi: 10.1016/j.funbio.2024.07.004 39653491

[B3] Bacsi Z. Jarso D. D. (2026). Cassava response to weather variability in eastern Africa. Agriculture 16, 209. doi: 10.3390/agriculture16020209 30654563

[B4] Bennacer A. Sahir-Halouane F. Alvarez M. Oukali Z. Bennacer N. E. H. Foughalia A. . (2025). *Talaromyces pinophilus* strain HD25G2 as a novel biocontrol agent of *Fusarium culmorum*, the causal agent of root and crown rot of soft wheat. J. Fungi 11, 588. doi: 10.3390/jof11080588 40863540 PMC12387219

[B5] Berg G. Köberl M. Rybakova D. Müller H. Grosch R. Smalla K. . (2017). Plant microbial diversity is suggested as the key to future biocontrol and health trends. FEMS Microbiol. Ecol. 93, fix050. doi: 10.1093/femsec/fix050 28430944

[B6] Berg G. Rybakova D. Fischer D. Cernava T. Vergès M. C. C. Charles T. . (2020). Microbiome definition re-visited: Old concepts and new challenges. Microbiome 8, 103. doi: 10.1186/s40168-020-00875-0 32605663 PMC7329523

[B7] Boas S. A. V. Oliveira S. A. S. D. Bragança C. A. D. Ramos J. B. Oliveira E. J. D. (2017). Survey of fungi associated with cassava root rot from different producing regions in Brazil. Sci. Agric. 74, 60–67. doi: 10.1590/0103-9016-2015-0366 41099703

[B8] Borku A. W. Tora T. T. Masha M. (2025). Cassava in focus: A comprehensive literature review, its production, processing landscape, and multi-dimensional benefits to society. Food Chem. Adv. 7, 100945. doi: 10.1016/j.focha.2025.100945 38826717

[B9] Bray R. H. Kurtz L. T. (1945). Determination of total, organic, and available forms of phosphorus in soils. Soil Sci. 59, 39–46. doi: 10.1097/00010694-194501000-00006 42348292

[B10] Bulgarelli D. Schlaeppi K. Spaepen S. Van Themaat E. V. L. Schulze-Lefert P. (2013). Structure and functions of the bacterial microbiota of plants. Annu. Rev. Plant Biol. 64, 807–838. doi: 10.1146/annurev-arplant-050312-120106 23373698

[B11] Cai J. Zhang J. Ding Y. Yu S. Lin H. Yuan Z. . (2021). Different fertilizers applied alter fungal community structure in rhizospheric soil of cassava (*Manihot esculenta* Crantz) and increase crop yield. Front. Microbiol. 12, 663781. doi: 10.3389/fmicb.2021.663781 34858357 PMC8631426

[B12] Chaudhari H. G. Prajapati S. Wardah Z. H. Raol G. Prajapati V. Patel R. . (2023). Decoding the microbial universe with metagenomics: A brief insight. Front. Genet. 14, 1119740. doi: 10.3389/fgene.2023.1119740 37197021 PMC10183756

[B13] Chen S. Zhou Y. Chen Y. Gu J. (2018). fastp: An ultra-fast all-in-one FASTQ preprocessor. Bioinformatics 34, i884–i890. doi: 10.1093/bioinformatics/bty560 30423086 PMC6129281

[B14] Compant S. Samad A. Faist H. Sessitsch A. (2019). A review on the plant microbiome: Ecology, functions, and emerging trends in microbial application. J. Adv. Res. 19, 29–37. doi: 10.1016/j.jare.2019.03.004 31341667 PMC6630030

[B15] Cruz D. R. Leandro L. F. Munkvold G. P. (2019). Effects of temperature and pH on *Fusarium oxysporum* and soybean seedling disease. Plant Dis. 103, 3234–3243. doi: 10.1094/pdis-11-18-1952-re 31573433

[B16] Darriaut R. Marzari T. Lailheugue V. Tran J. Martins G. Marguerit E. . (2024). Microbial dysbiosis in roots and rhizosphere of grapevines experiencing decline is associated with active metabolic functions. Front. Plant Sci. 15, 1358213. doi: 10.3389/fpls.2024.1358213 38628369 PMC11018932

[B17] da Silva J. S. A. da Silva Santos A. C. de Souza C. A. F. da Costa D. P. de Queiroz Brito A. C. do Nascimento Barbosa R. . (2025). First reports of *Fusarium agrestense*, *F. gossypinum*, *F. grosmichelii* and *F. triseptatum* causing cassava root rot in Pernambuco, Brazil. Crop Prot. 187, 106947. doi: 10.1016/j.cropro.2024.106947 38826717

[B18] Dastogeer K. G. Hasan M. S. Niloy S. M. H. Khatun M. M. Alve S. M. T. I. Zaman S. B. . (2026). Fungal endophytes in biocontrol of plant fungal pathogens: Current landscape, mechanisms, drivers, bottlenecks, and future directions. Curr. Plant Biol. 48, 100640. doi: 10.1016/j.cpb.2026.100640 38826717

[B19] De Cal A. Sztejnberg A. Sabuquillo P. Melgarejo P. (2009). Management of Fusarium wilt on melon and watermelon by Penicillium oxalicum. Biol. Control 51, 480–486. doi: 10.1016/j.biocontrol.2009.08.011 38826717

[B20] Dhariwal A. Chong J. Habib S. King I. L. Agellon L. B. Xia J. (2017). MicrobiomeAnalyst: A web-based tool for comprehensive statistical, visual and meta-analysis of microbiome data. Nucleic Acids Res. 45, W180–W188. doi: 10.1093/nar/gkx295 28449106 PMC5570177

[B21] Dong S. Zhang B. Hou W. Zhou X. Gao Q. (2024). Differential effects of sulfur fertilization on soil microbial communities and maize yield enhancement. Agronomy 14, 2251. doi: 10.3390/agronomy14102251 30654563

[B22] Du Y. Han X. Tsuda K. (2025). Microbiome-mediated plant disease resistance: Recent advances and future directions. J. Gen. Plant Pathol. 91, 1–17. doi: 10.1007/s10327-024-01204-1 30311153

[B23] Edwards J. Johnson C. Santos-Medellín C. Lurie E. Podishetty N. K. Bhatnagar S. . (2015). Structure, variation, and assembly of the root-associated microbiomes of rice. Proc. Natl. Acad. Sci. U.S.A. 112, E911–E920. doi: 10.1073/pnas.1414592112 25605935 PMC4345613

[B24] Elkhairy B. M. Salama N. M. Desouki A. M. Abdelrazek A. B. Soliman K. A. Ibrahim S. A. . (2023). Towards unlocking the biocontrol potential of Pichia kudriavzevii for plant fungal diseases: *in vitro* and *in vivo* assessments with candidate secreted protein prediction. BMC Microbiol. 23, 356. doi: 10.1186/s12866-023-03047-w 37980509 PMC10657120

[B25] Food and Agriculture Organization (2022). Faostat. Available online at: https://www.fao.org/faostat/en/ (Accessed December 26, 2025).

[B26] Gangaraj R. Kundu A. Rana V. S. Das A. Chawla G. Prakash G. . (2023). Metabolomic profiling and its association with the bio-efficacy of Aspergillus Niger strain against Fusarium wilt of guava. Front. Microbiol. 14, 1142144. doi: 10.3389/fmicb.2023.1142144 37168123 PMC10165087

[B27] Gao J. Dong A. Li J. Xu J. Liang Z. Logrieco A. F. (2025). Exploring fungal communication mechanisms in the rhizosphere microbiome for a sustainable green agriculture. J. Fungi 11, 726. doi: 10.3390/jof11100726 41149915 PMC12565245

[B28] Gao Z. Han M. Hu Y. Li Z. Liu C. Wang X. . (2019). Effects of continuous cropping of sweet potato on the fungal community structure in rhizospheric soil. Front. Microbiol. 10, 2269. doi: 10.3389/fmicb.2019.02269 31632375 PMC6783561

[B29] Government of India (2023). Agricultural Statistics at a Glance 2023. Available online at: http://desagri.gov.in (Accessed December 26, 2025).

[B30] Guo Q. Yu Y. Zhang S. Guan Y. Jiang N. Pang S. . (2024). Core bacteria and fungi in response to residue retention and their contribution to soil multifunctionality in maize agroecosystem. Sci. Total Environ. 921, 171191. doi: 10.1016/j.scitotenv.2024.171191 38402993

[B31] Gupta U. C. (1967). A simplified method for determining hot-water-soluble boron in podzol soils. Soil Sci. 103, 424–428. doi: 10.1097/00010694-196706000-00009 42348292

[B32] Gupta A. Singh U. B. Sahu P. K. Paul S. Kumar A. Malviya D. . (2022). Linking soil microbial diversity to modern agriculture practices: A review. Int. J. Environ. Res. Public Health 19, 3141. doi: 10.3390/ijerph19053141 35270832 PMC8910389

[B33] Harman G. E. Howell C. R. Viterbo A. Chet I. Lorito M. (2004). Trichoderma species—opportunistic, avirulent plant symbionts. Nat. Rev. Microbiol. 2, 43–56. doi: 10.1038/nrmicro797 15035008

[B34] Hisham M. M. Chandran J. Gopinath P. P. (2025). RAISINS: Integrating R and AI for agricultural data analysis. J. Sustain. Technol. Agric. 1 (1). doi: 10.5281/zenodo.15622128 42270437

[B35] Hohenfeld C. S. Passos A. R. de Carvalho H. W. L. de Oliveira S. A. S. de Oliveira E. J. (2022). Genome-wide association study and selection for field resistance to cassava root rot disease and productive traits. PloS One 17, e0270020. doi: 10.1371/journal.pone.0270020 35709238 PMC9202857

[B36] Hooks K. B. O’Malley M. A. (2017). Dysbiosis and its discontents. mBio 8, e01492-17. doi: 10.1128/mbio.01492-17 29018121 PMC5635691

[B37] Hossain M. M. Sultana F. Mostafa M. Rubayet M. T. Mishu N. J. Khan I. . (2025). Biological management of soil-borne pathogens through tripartite rhizosphere interactions with plant growth-promoting fungi. Appl. Microbiol. 5, 123. doi: 10.3390/applmicrobiol5040123 30654563

[B38] Immanuel S. Jaganathan D. Prakash P. Sivakumar P. S. (2024). Cassava for food security, poverty reduction and climate resilience: A review. Indian J. Ecol. 51, 21–31. doi: 10.55362/IJE/2024/4191

[B39] Iqbal A. Rehman M. M. U. Sajjad W. Degen A. A. Rafiq M. Jiahuan N. . (2024). Patterns of bacterial communities in the rhizosphere and rhizoplane of alpine wet meadows. Environ. Res. 241, 117672. doi: 10.1016/j.envres.2023.117672 37980986

[B40] Jackson M. L. (1973). Soil Chemical Analysis (New Delhi: Prentice Hall of India Ltd).

[B41] Jakuschkin B. Fievet V. Schwaller L. Fort T. Robin C. Vacher C. (2016). Deciphering the pathobiome: Intra- and interkingdom interactions involving the pathogen Erysiphe alphitoides. Microb. Ecol. 72, 870–880. doi: 10.1007/s00248-016-0777-x 27147439

[B42] Jeeva M. L. Veena S. S. John K. S. Mathew J. Makeshkumar T. Arutselvan R. . (2025). “ Cassava stem and root rot management: Bridging the gap between research and farmer adoption,” in Book of Abstracts: International Symposium on Tropical Root & Tuber Crops for Nutrition, Agri-Food Systems, Resilience, Entrepreneurship and Sustainability (ISTRTC 4 NARES) ( ICAR-Central Tuber Crops Research Institute, Thiruvananthapuram), 202.

[B43] Jeeva M. L. Veena S. S. Makeshkumar T. Karthikeyan S. Amrutha P. R. Shilpa S. U. (2020). Emerging cassava root and stem rot: A challenge to wetland farmers in Kerala. J. Root Crops 46, 114–117.

[B44] Jing T. Li K. Wang L. Eissa M. A. Cai B. Yun T. . (2025). Acidification and nutrient imbalances drive Fusarium wilt severity in banana (Musa spp.) grown on tropical latosols. J. Fungi 11, 611. doi: 10.3390/jof11090611 41003157 PMC12470300

[B45] Kala C. Gangopadhyay S. Goden S. L. (2016). Ecofriendly management of wilt caused by *Fusarium oxysporum f. sp. ciceri* in chickpea. Legume Research 39 (1), 129–134. doi: 10.18805/lr.v0iof.6789

[B46] Karlström A. Calle F. Salazar S. Morante N. Dufour D. Ceballos H. (2016). Biological implications in cassava for the production of amylose-free starch: Impact on root yield and related traits. Front. Plant Sci. 7, 604. doi: 10.3389/fpls.2016.00604 27242813 PMC4873506

[B47] Kaushal M. Swennen R. Mahuku G. (2020). Unlocking the microbiome communities of banana (Musa spp.) under disease stressed (Fusarium wilt) and non-stressed conditions. Microorganisms 8, 443. doi: 10.3390/microorganisms8030443 32245146 PMC7144012

[B48] Kowalska J. Krzymińska J. Tyburski J. (2022). Yeasts as a potential biological agent in plant disease protection and yield improvement—A short review. Agriculture 12, 1404. doi: 10.3390/agriculture12091404 30654563

[B49] Kumar M. Ansari W. A. Zeyad M. T. Singh A. Chakdar H. Kumar A. . (2023). Core microbiota of wheat rhizosphere under Upper Indo-Gangetic plains and their response to soil physicochemical properties. Front. Plant Sci. 14, 1186162. doi: 10.3389/fpls.2023.1186162 37255554 PMC10226189

[B50] Lareen A. Burton F. Schäfer P. (2016). Plant root-microbe communication in shaping root microbiomes. Plant Mol. Biol. 90, 575–587. doi: 10.1007/s11103-015-0417-8 26729479 PMC4819777

[B51] Lem A. C. Annih M. G. Kinge T. R. (2025). Pathogenicity and effects of soil physicochemical properties on fungal disease incidence and severity of cassava (Manihot esculenta Crantz.) in Bamenda, Cameroon. J. Appl. Biosci. 212, 22462–22485.

[B52] Lex A. Gehlenborg N. Strobelt H. Vuillemot R. Pfister H. (2014). UpSet: Visualization of intersecting sets. IEEE Trans. Vis. Comput. Graph. 20, 1983–1992. doi: 10.1109/TVCG.2014.2346248 26356912 PMC4720993

[B53] Li X. Chen D. Carrión V. J. Revillini D. Yin S. Dong Y. . (2023). Acidification suppresses the natural capacity of soil microbiome to fight pathogenic Fusarium infections. Nat. Commun. 14, 5090. doi: 10.1038/s41467-023-40810-z 37607924 PMC10444831

[B54] Li P. Dini-Andreote F. Jiang J. (2024). Exploiting microbial competition to promote plant health. Trends Plant Sci. 29, 1056–1058. doi: 10.1016/j.tplants.2024.05.003 38760241

[B55] Li J. Hu H. W. Cai Z. J. Lei Y. R. Zhang M. Y. Li W. . (2018). Contrasting soil bacterial and fungal communities between the swamp and upland in the boreal forest and their biogeographic distribution patterns. Wetlands 38, 1101–1111. doi: 10.1007/s13157-018-1066-x 30311153

[B56] Li J. Wang C. Liang W. Liu S. (2021). Rhizosphere microbiome: The emerging barrier in plant-pathogen interactions. Front. Microbiol. 12, 772420. doi: 10.3389/fmicb.2021.772420 34777326 PMC8586421

[B57] Liang S. Li H. Wu H. Yan B. Song A. (2023). Microorganisms in coastal wetland sediments: A review on microbial community structure, functional gene, and environmental potential. Front. Microbiol. 14, 1163896. doi: 10.3389/fmicb.2023.1163896 37333635 PMC10272453

[B58] Liao Q. Shen Z. Li G. Peng X. Peng X. Liao Q. . (2026). Soil microbial community stability: A key to sustainable cassava production. Biol. Fertil. Soils 62, 607–628. doi: 10.1007/s00374-026-02000-z 30311153

[B59] Liu Q. Lu X. Xiang C. Yu S. Zhang J. Li K. . (2025). The effect of different types of fertilizers on the growth of cassava and the fungal community in rhizosphere soil. J. Fungi 11, 235. doi: 10.3390/jof11030235 40137271 PMC11943314

[B60] Liu N. Luo X. Tian Y. Lai D. Zhang L. Lin F. . (2019). The stereoisomeric Bacillus subtilis HN09 metabolite 3,4-dihydroxy-3-methyl-2-pentanone induces disease resistance in Arabidopsis via different signalling pathways. BMC Plant Biol. 19, 384. doi: 10.1186/s12870-019-1985-6 31488058 PMC6727425

[B61] Luo M. Chen Y. He J. Tang X. Wu X. Xu C. (2021). Identification of a new Talaromyces strain DYM25 isolated from the Yap Trench as a biocontrol agent against Fusarium wilt of cucumber. Microbiol. Res. 251, 126841. doi: 10.1016/j.micres.2021.126841 34385083

[B62] Ma L. Bai Y. Li X. Li J. Li F. Yue Y. . (2025). Compartment-specific core microbiomes and functions in rice. Plant Soil 1, 1–20. doi: 10.1007/s11104-025-07558-5 30311153

[B63] Ma L. J. Geiser D. M. Proctor R. H. Rooney A. P. O'Donnell K. Trail F. . (2013). Fusarium pathogenomics. Annu. Rev. Microbiol. 67, 399–416. doi: 10.1146/annurev-micro-092412-155650 24024636

[B64] Massoumi A. Cornfield A. H. (1963). A rapid method for determining sulphate in water extracts of soils. Analyst 88, 321–322. doi: 10.1039/AN9638800321

[B65] Maurice K. Bourceret A. Youssef S. Boivin S. Laurent-Webb L. Damasio C. . (2024). Anthropic disturbances impact the soil microbial network structure and stability to a greater extent than natural disturbances in an arid ecosystem. Sci. Total Environ. 907, 167969. doi: 10.1016/j.scitotenv.2023.167969 37914121

[B66] McCallum E. J. Anjanappa R. B. Gruissem W. (2017). Tackling agriculturally relevant diseases in the staple crop cassava (Manihot esculenta). Curr. Opin. Plant Biol. 38, 50–58. doi: 10.1016/j.pbi.2017.04.008 28477536

[B67] McMurdie P. J. Holmes S. (2013). phyloseq: an R package for reproducible interactive analysis and graphics of microbiome census data. PloS One 8, e61217. doi: 10.1371/journal.pone.0061217 23630581 PMC3632530

[B68] Mendes R. Garbeva P. Raaijmakers J. M. (2013). The rhizosphere microbiome: significance of plant beneficial, plant pathogenic, and human pathogenic microorganisms. FEMS Microbiol. Rev. 37, 634–663. doi: 10.1111/1574-6976.12028 23790204

[B69] Moore E. Arnscheidt A. Krüger A. Strömpl C. Mau M. (2004). “ Simplified protocols for the preparation of genomic DNA from bacterial cultures,” in Molecular Microbial Ecology Manual. Eds. Akkermans A. D. L. van Elsas J. D. de Bruijn F. J. ( Kluwer Academic Publishers, Dordrecht), 1–15.

[B70] Oksanen J. Simpson G. L. Blanchet F. G. Kindt R. Legendre P. Minchin P. R. . (2025). Vegan: Community Ecology Package. Available online at: https://CRAN.R-project.org/package=vegan (Accessed December 26, 2025). R package version 2.7-1.

[B71] Orr R. Dennis P. G. Wong Y. Browne D. J. Cooper M. Birt H. W. . (2022). Nitrogen fertilizer rate but not form affects the severity of Fusarium wilt in banana. Front. Plant Sci. 13, 907819. doi: 10.3389/fpls.2022.907819 35941941 PMC9356348

[B72] Otekunrin O. A. (2024). Cassava (Manihot esculenta Crantz): a global scientific footprint—production, trade, and bibliometric insights. Discover Agric. 2, 94. doi: 10.1007/s44279-024-00121-3 30311153

[B73] Parray J. A. Egamberdieva D. Abd_Allah E. F. Sayyed R. Z. (2023). Soil microbiome metabolomics: a way forward to sustainable intensification. Front. Sustain. Food Syst. 7, 1251054. doi: 10.3389/fsufs.2023.1251054

[B74] Patel D. Patel A. Patel M. Goswami D. (2021). Talaromyces pinophilus strain M13: a portrayal of novel groundbreaking fungal strain for phytointensification. Environ. Sci. pollut. Res. 28, 8758–8769. doi: 10.1007/s11356-020-11152-w 33067792

[B75] Pezeshki S. R. DeLaune R. D. (2012). Soil oxidation-reduction in wetlands and its impact on plant functioning. Biology 1, 196–221. doi: 10.3390/biology1020196 24832223 PMC4009779

[B76] Raaijmakers J. M. Paulitz T. C. Steinberg C. Alabouvette C. Moënne-Loccoz Y. (2009). The rhizosphere: a playground and battlefield for soilborne pathogens and beneficial microorganisms. Plant Soil 321, 341–361. doi: 10.1007/s11104-008-9568-6 30311153

[B77] Raja H. A. Miller A. N. Pearce C. J. Oberlies N. H. (2017). Fungal identification using molecular tools: a primer for the natural products research community. J. Nat. Prod. 80, 756–770. doi: 10.1021/acs.jnatprod.6b01085 28199101 PMC5368684

[B78] Rini C. R. Sulochana K. K. (2007). Substrate evaluation for multiplication of Trichoderma spp. J. Trop. Agric. 45, 55–57.

[B79] Samadli H. (2026). The role of microorganisms in ecosystems and the maintenance of ecosystem stability. Nat. Sci. 8, 58–66. doi: 10.36719/2707-1146/64/58-66 42397674

[B80] Sangpueak R. Duchanee S. Saengchan C. Papathoti N. K. Hoang N. H. Thanh T. L. . (2023). Identification of cassava black stem and root rot agents in Thailand. Chil. J. Agric. Res. 83, 70–82. doi: 10.4067/S0718-58392023000100070

[B81] Scaria S. S. Balasubramanian B. Meyyazhagan A. Gangwar J. Jaison J. P. Kurian J. T. . (2024). Cassava (Manihot esculenta Crantz)—A potential source of phytochemicals, food, and nutrition—An updated review. eFood 5, e127. doi: 10.1002/efd2.127 41531421

[B82] Schoch C. L. Seifert K. A. Huhndorf S. Robert V. Spouge J. L. Levesque C. A. . (2012). Nuclear ribosomal internal transcribed spacer (ITS) region as a universal DNA barcode marker for Fungi. Proc. Natl. Acad. Sci. U.S.A. 109, 6241–6246. doi: 10.1073/pnas.1117018109 22454494 PMC3341068

[B83] Shade A. Handelsman J. (2012). Beyond the Venn diagram: the hunt for a core microbiome. Environ. Microbiol. 14, 4–12. doi: 10.1111/j.1462-2920.2011.02585.x 22004523

[B84] Silva I. O. da Rocha Amorim E. P. Junior N. A. N. Carnauba J. P. de Araújo Neto V. F. Peixinho G. S. (2022). The first report about Fusarium falciforme that causes root rot in cassava cv Rosinha, in Alagoas state, Brazil. Research Soc. Dev. 11, e221111234369-e221111234369. doi: 10.33448/rsd-v11i12.34369

[B85] Sims J. T. Johnson G. V. (1991). “ Micronutrient soil tests,” in Micronutrients in Agriculture. Eds. Mortvedt J. J. Cox F. R. Shuman L. M. Welch R. M. ( Soil Science Society of America Book Series No. 4, Madison, WI), 427–476. doi: 10.2136/sssabookser4.2ed.c12

[B86] Skidmore A. M. Dickinson C. H. (1976). Colony interactions and hyphal interference between Septoria nodorum and phylloplane fungi. Trans. Br. Mycol. Soc 66, 57–64. doi: 10.1016/s0007-1536(76)80092-7

[B87] Subbiah B. V. Asija G. L. (1956). A rapid procedure for the determination of available nitrogen in soil. Curr. Sci. 25, 259–260.

[B88] Subhash A. P. Veena S. S. Makeshkumar T. Anith K. N. (2025). Piriformospora indica and Arbuscular Mycorrhizal Fungus suppress fungal root rot and mosaic diseases of cassava. Symbiosis 95, 241–254. doi: 10.1007/s13199-025-01044-3 30311153

[B89] Tao J. Jin J. Lu P. Yu S. Gu M. Wang J. . (2025). Bacterial wilt disease alters the structure and function of fungal communities around plant roots. BMC Plant Biol. 25, 39. doi: 10.1186/s12870-025-06056-1 39789485 PMC11721222

[B90] Teixeira P. J. P. Colaianni N. R. Fitzpatrick C. R. Dangl J. L. (2019). Beyond pathogens: microbiota interactions with the plant immune system. Curr. Opin. Microbiol. 49, 7–17. doi: 10.1016/j.mib.2019.08.003 31563068

[B91] Thomas V. E. Abeysinghe G. Goetze P. K. Jo Y. K. Antony-Babu S. (2025). The distinguished microbiome: differentiating microbial dysbiosis and the pathobiome of banana Fusarium tropical Race 4. Phytobiomes J. 9, 608–619. doi: 10.1094/PBIOMES-10-24-0097-R

[B92] Trivedi P. Leach J. E. Tringe S. G. Sa T. Singh B. K. (2020). Plant–microbiome interactions: from community assembly to plant health. Nat. Rev. Microbiol. 18, 607–621. doi: 10.1038/s41579-020-0412-1 32788714

[B93] Vincent J. M. (1947). Distortion of fungal hyphae in the presence of certain inhibitors. Nature 159, 850. doi: 10.1038/159850b0 20343980

[B94] Walkley A. Black I. A. (1934). An examination of the Degtjareff method for determining soil organic matter, and a proposed modification of the chromic acid titration method. Soil Sci. 37, 29–38. doi: 10.1097/00010694-193401000-00003 42348292

[B95] Wang T. Chen Y. Yan M. Wang H. Guo K. Zhou X. . (2025). Amplicon sequencing reveals rhizosphere fungal dysbiosis facilitates Goji berry root rot onset. Plants 14, 3325. doi: 10.3390/plants14213325 41225874 PMC12610515

[B96] Wang S. Ma T. Yao X. Yao Z. Wang Z. Dong Z. . (2023). Structure of endophytes in the root, stem, and leaf tissues of sweetpotato and their response to sweetpotato scab disease caused by Elsinoë batatas. Agronomy 13, 2965. doi: 10.3390/agronomy13122965 30654563

[B97] Wei Z. Gu Y. Friman V. P. Kowalchuk G. A. Xu Y. Shen Q. . (2019). Initial soil microbiome composition and functioning predetermine future plant health. Sci. Adv. 5, eaaw0759. doi: 10.1126/sciadv.aaw0759 31579818 PMC6760924

[B98] Wickham H. (2016). Ggplot2: Elegant Graphics for Data Analysis (Cham: Springer International Publishing). doi: 10.1007/978-3-319-24277-4

[B99] Wingett S. W. Andrews S. (2018). FastQ Screen: A tool for multi-genome mapping and quality control. F1000Res 7, 1338. doi: 10.12688/f1000research.15931.2 30254741 PMC6124377

[B100] Wood D. E. Lu J. Langmead B. (2019). Improved metagenomic analysis with Kraken 2. Genome Biol. 20, 257. doi: 10.1186/s13059-019-1891-0 31779668 PMC6883579

[B101] Wu Z. Chen J. Chen J. Yang Y. Zhou A. Wu J . (2025). The relationship between pomegranate root collar rot and the diversity of fungal communities in its rhizosphere. Front. Microbiol. 16, 1573724. doi: 10.3389/fmicb.2025.1573724 40190735 PMC11968712

[B102] Wu C. Liu H. Carvalhais L. C. Guo J. Cai P. Zhong J. . (2026). Root exudate‐associated microbiome assembly contributes to viral disease resistance in wheat. New Phytol. 251 (3), 1397–1414. doi: 10.1111/nph.71254 42136370

[B103] Yan Z. Yang S. Chen L. Zou Y. Zhao Y. Yan G. . (2024). Responses of soil fungal community composition and function to wetland degradation in the Songnen Plain, northeastern China. Front. Plant Sci. 15, 1441613. doi: 10.3389/fpls.2024.1441613 39315367 PMC11416943

[B104] Yin J. (2026). Mechanistic insights for microbiome application in plant disease resistance. Mol. Plant Pathol. 27, e70233. doi: 10.1111/mpp.70233 41793016 PMC12966624

[B105] Zhang K. Wang L. Si H. Guo H. Liu J. Jia J. . (2022). Maize stalk rot caused by Fusarium graminearum alters soil microbial composition and is directly inhibited by Bacillus siamensis isolated from rhizosphere soil. Front. Microbiol. 13, 986401. doi: 10.3389/fmicb.2022.986401 36338067 PMC9630747

[B106] Zhang L. Zhang J. Wei Y. Hu W. Liu G. Zeng H. . (2021). Microbiome‐wide association studies reveal correlations between the structure and metabolism of the rhizosphere microbiome and disease resistance in cassava. Plant Biotechnol. J. 19, 689–701. doi: 10.1111/pbi.13495 33095967 PMC8051613

[B107] Zhou Y. Liu D. Li F. Dong Y. Jin Z. Liao Y. . (2024). Superiority of native soil core microbiomes in supporting plant growth. Nat. Commun. 15, 6599. doi: 10.1038/s41467-024-50685-3 39097606 PMC11297980

