# Supplementary material for: Integrated metabarcoding and culturomics reveal the rot-suppressive rhizoplane mycobiome of cassava across contrasting agroecological habitats
Source: Front Plant Sci. 2026 Aug 5;17:1782244. doi: 10.3389/fpls.2026.1782244 (PMC13485920; doi:10.3389/fpls.2026.1782244)
Supplement: Supplementary file 1 [file Table1.docx]

**Supplementary Table 1. Analysis of Soil Physicochemical Properties**

| **Parameter** | **Wetland soil** | **Upland soil** |
| --- | --- | --- |
| pH | 5.49 | 6.85 |
| EC (dS/m) | 0.11 | 0.10 |
| OC (%) | 1.81 | 1.68 |
| N (kg/ha) | 288.51 | 263.42 |
| P (kg/ha) | 186.10 | 84.00 |
| K (kg/ha) | 220.0 | 297.0 |
| B (ppm) | 0.54 | 0.51 |
| S (ppm) | 80.5 | 29.5 |
| Ca (ppm) | 380.0 | 540.0 |
| Mg (ppm) | 144.0 | 144.0 |
| Fe (ppm) | 29.4 | 31.4 |
| Mn (ppm) | 21.2 | 22.8 |
| Zn (ppm) | 1.8 | 1.7 |
| Cu (ppm) | 2.2 | 1.5 |

**Supplementary Table 2. Relative Abundance and Dynamics of Total Fungal Communities in Cassava Rhizoplane at Family Level**

| **Taxa** | **Upland** | | **Wetland Diseased** | | **Wetland Healthy** | |
| --- | --- | --- | --- | --- | --- | --- |
|  | **Abundance** | **Per cent** | **Abundance** | **Per cent** | **Abundance** | **Per cent** |
| **Aspergillaceae** | 12969 | 0.06 | 14750 | 0.04 | 19939 | 0.19 |
| **Chaetomiaceae** | 38 | 0.00 | 845 | 0.00 | 338 | 0.00 |
| **Clavicipitaceae** | 4 | 0.00 | 25 | 0.00 | 139 | 0.00 |
| **Cryptococcaceae** | 394 | 0.00 | 358 | 0.00 | 1085 | 0.01 |
| **Debaryomycetaceae** | 8256 | 0.04 | 2253 | 0.01 | 429 | 0.00 |
| **Glomerellaceae** | 79903 | 0.35 | 21 | 0.00 | 49 | 0.00 |
| **Hypocreaceae** | 135 | 0.00 | 982 | 0.00 | 480 | 0.00 |
| **Malasseziaceae** | 126 | 0.00 | 527 | 0.00 | 43 | 0.00 |
| **Marasmiaceae** | 99 | 0.00 | 345 | 0.00 | 4273 | 0.04 |
| **Metschnikowiaceae** | 303 | 0.00 | 1323 | 0.00 | 1124 | 0.01 |
| **Mycosphaerellaceae** | 31346 | 0.14 | 65 | 0.00 | 95 | 0.00 |
| **Nectriaceae** | 12027 | 0.05 | 3558 | 0.01 | 9215 | 0.09 |
| **Pichiaceae** | 9918 | 0.04 | 93024 | 0.24 | 22672 | 0.21 |
| **Pleosporineae** | 55966 | 0.25 | 2915 | 0.01 | 6217 | 0.06 |
| **Pucciniaceae** | 1512 | 0.01 | 451 | 0.00 | 1133 | 0.01 |
| **Saccharomycetaceae** | 1736 | 0.01 | 1979 | 0.01 | 3743 | 0.04 |
| **Saccharomycodaceae** | 202 | 0.00 | 251 | 0.00 | 217 | 0.00 |
| **Schizosaccharomycetaceae** | 21 | 0.00 | 81 | 0.00 | 422 | 0.00 |
| **Sclerotiniaceae** | 196 | 0.00 | 484 | 0.00 | 318 | 0.00 |
| **Sordariaceae** | 97 | 0.00 | 114 | 0.00 | 418 | 0.00 |
| **Strophariaceae** | 584 | 0.00 | 15095 | 0.04 | 6039 | 0.06 |
| **Trichocomaceae** | 9033 | 0.04 | 1836 | 0.00 | 1793 | 0.02 |
| **Trichomonascaceae** | 1482 | 0.01 | 246066 | 0.63 | 25923 | 0.24 |
| **Trichosporonaceae** | 730 | 0.00 | 70 | 0.00 | 153 | 0.00 |
| **Ustilaginaceae** | 5 | 0.00 | 100 | 0.00 | 17 | 0.00 |

**Supplementary Table 3. Relative Abundance and Dynamics of Total Fungal Communities in Cassava Rhizoplane at Genus Level**

| **Taxa** | **Upland** | | **Wetland Diseased** | | **Wetland Healthy** | |
| --- | --- | --- | --- | --- | --- | --- |
|  | **Abundance** | **Per cent** | **Abundance** | **Per cent** | **Abundance** | **Per cent** |
| ***Ascochyta*** | 55966 | 0.25 | 2915 | 0.01 | 6217 | 0.06 |
| ***Aspergillus*** | 11469 | 0.05 | 8592 | 0.02 | 12310 | 0.12 |
| ***Botrytis*** | 196 | 0.00 | 484 | 0.00 | 318 | 0.00 |
| ***Brettanomyces*** | 317 | 0.00 | 265 | 0.00 | 317 | 0.00 |
| ***Candida*** | 6894 | 0.03 | 1368 | 0.00 | 1153 | 0.01 |
| ***Colletotrichum*** | 79903 | 0.35 | 21 | 0.00 | 49 | 0.00 |
| ***Cryptococcus*** | 394 | 0.00 | 358 | 0.00 | 1085 | 0.01 |
| ***Cutaneotrichosporon*** | 730 | 0.00 | 70 | 0.00 | 153 | 0.00 |
| ***Eremothecium*** | 80 | 0.00 | 620 | 0.00 | 385 | 0.00 |
| ***Fulvia*** | 31132 | 0.14 | 55 | 0.00 | 84 | 0.00 |
| ***Fusarium*** | 12027 | 0.05 | 3558 | 0.01 | 9215 | 0.09 |
| ***Kluyveromyces*** | 201 | 0.00 | 91 | 0.00 | 224 | 0.00 |
| ***Malassezia*** | 126 | 0.00 | 527 | 0.00 | 43 | 0.00 |
| ***Marasmius*** | 99 | 0.00 | 345 | 0.00 | 4273 | 0.04 |
| ***Nakaseomyces*** | 856 | 0.00 | 598 | 0.00 | 404 | 0.00 |
| ***Neurospora*** | 97 | 0.00 | 114 | 0.00 | 418 | 0.00 |
| ***Ogataea*** | 130 | 0.00 | 753 | 0.00 | 157 | 0.00 |
| ***Penicillium*** | 1500 | 0.01 | 6158 | 0.02 | 7629 | 0.07 |
| ***Pichia*** | 9471 | 0.04 | 92006 | 0.24 | 22198 | 0.21 |
| ***Pochonia*** | 4 | 0.00 | 25 | 0.00 | 139 | 0.00 |
| ***Psilocybe*** | 584 | 0.00 | 15095 | 0.04 | 6039 | 0.06 |
| ***Puccinia*** | 1512 | 0.01 | 451 | 0.00 | 1133 | 0.01 |
| ***Saccharomycodes*** | 202 | 0.00 | 251 | 0.00 | 217 | 0.00 |
| ***Scheffersomyces*** | 1665 | 0.01 | 2208 | 0.01 | 400 | 0.00 |
| ***Schizosaccharomyces*** | 21 | 0.00 | 81 | 0.00 | 422 | 0.00 |
| ***Sporisorium*** | 5 | 0.00 | 100 | 0.00 | 17 | 0.00 |
| ***Sugiyamaella*** | 1482 | 0.01 | 246066 | 0.63 | 25923 | 0.24 |
| ***Talaromyces*** | 9033 | 0.04 | 1836 | 0.00 | 1793 | 0.02 |
| ***Tetrapisispora*** | 448 | 0.00 | 291 | 0.00 | 1014 | 0.01 |
| ***Thermothelomyces*** | 22 | 0.00 | 121 | 0.00 | 32 | 0.00 |
| ***Thermothielavioides*** | 16 | 0.00 | 724 | 0.00 | 306 | 0.00 |
| ***Torulaspora*** | 0 | 0.00 | 5 | 0.00 | 505 | 0.00 |
| ***Trichoderma*** | 135 | 0.00 | 982 | 0.00 | 480 | 0.00 |
| ***Zygosaccharomyces*** | 151 | 0.00 | 374 | 0.00 | 1211 | 0.01 |
| ***Zymoseptoria*** | 214 | 0.00 | 10 | 0.00 | 11 | 0.00 |
